# Splenic macrophages are required for protective innate immunity against West Nile virus

**DOI:** 10.1371/journal.pone.0191690

**Published:** 2018-02-06

**Authors:** Marianne A. Bryan, Daniela Giordano, Kevin E. Draves, Richard Green, Michael Gale, Edward A. Clark

**Affiliations:** 1 Department of Immunology, University of Washington, Seattle, WA, United States of America; 2 The Center for Innate Immunity and Immune Disease, University of Washington, Seattle, WA, United States of America; Washington University, UNITED STATES

## Abstract

Although the spleen is a major site for West Nile virus (WNV) replication and spread, relatively little is known about which innate cells in the spleen replicate WNV, control viral dissemination, and/or prime innate and adaptive immune responses. Here we tested if splenic macrophages (MΦs) were necessary for control of WNV infection. We selectively depleted splenic MΦs, but not draining lymph node MΦs, by injecting mice intravenously with clodronate liposomes several days prior to infecting them with WNV. Mice missing splenic MΦs succumbed to WNV infection after an increased and accelerated spread of virus to the spleen and the brain. WNV-specific Ab and CTL responses were normal in splenic MΦ-depleted mice; however, numbers of NK cells and CD4 and CD8 T cells were significantly increased in the brains of infected mice. Splenic MΦ deficiency led to increased WNV in other splenic innate immune cells including CD11b^-^ DCs, newly formed MΦs and monocytes. Unlike other splenic myeloid subsets, splenic MΦs express high levels of mRNAs encoding the complement protein C1q, the apoptotic cell clearance protein Mertk, the IL-18 cytokine and the FcγR1 receptor. Splenic MΦ-deficient mice may be highly susceptible to WNV infection in part to a deficiency in C1q, Mertk, IL-18 or Caspase 12 expression.

## Introduction

West Nile virus (WNV) is a positive-stranded, enveloped, RNA flavivirus and is a member of the Flavivirus genus that are usually transmitted by mosquitos; some members such as the closely-related Japanese encephalitis virus cause viral encephalitis, and central nervous system (CNS) infection (Zika virus; ZIKV) while other members such as dengue virus, yellow fever virus and ZIKV are also associated with systemic diseases [1]. After its introduction into the New York area in 1999, WNV spread rapidly around the country and into North and South America [2]. It is now endemic in all continents except Antarctica, and its virulence is underscored by the large outbreak in the United States in 2012, where over 5000 people were infected, half of which had neurologic disease [3, 4]. Currently, there is neither a preventative vaccine nor an effective antiviral treatment for WNV [5, 6].

Both innate and adaptive immune responses are required for controlling WNV infections [7,8]. WNV is recognized by innate immune pattern recognition receptors (PRR) including the intracellular RNA sensors retinoic acid-inducible gene 1 (RIG-I) and melanoma differentiation-associated gene 5 (MDA5) and endosomal RNA sensors TLR3 and TLR7, and, like other flaviviruses, employs a number of strategies to counteract its recognition [7–9]. WNV is transmitted to humans s.c. through the bites of infected *Culex* species mosquitoes. An animal model of infection where mice are inoculated with WNV in a footpad mimics the natural inoculation route of WNV and has facilitated the characterization of how innate and adaptive immune responses develop after WNV infection and contribute to protective immunity [7, 10]. For example, infection of mice deficient in the adaptor downstream of RIG-I and MDA5, mitochondrial antiviral signaling (MAVS), revealed that RIG-I-like receptors and MAVS are essential for innate protective immunity against WNV [11]. Early control of WNV infection also depends on type 1 and type 3 IFN responses. Type I IFN receptor (IFNAR)-deficient (IFNAR^-/-^) mice infected with WNV all die within 4 days post-infection (p.i.) with doses of WNV that produce little or no death in wildtype (WT) mice [10], while mice lacking the type 3 IFN receptor chain IL-28Rα, exhibit increased CNS infection concomitant with loss of blood brain barrier integrity [12]. Similar studies using knockout (KO) mice showed that TLR7 and its adaptor protein MyD88 as well as cytokines like IL-12p40 and IL-23 are required for protection against WNV [13, 14].

While these and other studies with KO mice have helped to identify key elements involved in global protection against WNV, it is less clear which immune cell types are required for innate immune responses to be most effective. Furthermore, the specific target cells in the spleen and other peripheral lymphoid tissues that are infected and/or respond to WNV are not that well defined. Dendritic cells (DCs) and macrophages (MΦs) can be infected by WNV and play a pivotal role in linking innate and adaptive immune responses [7, 15]. A recent study using conditional KO mice showed that expression of IFNAR in DCs and in myeloid cells including MΦs, monocytes (MOs) and neutrophils (Nphs) is required for protection against WNV [16]. Systemic depletion of monocytes and MΦs via injection of clodronate liposomes (CLLs) resulted in greatly accelerated encephalitis and death after WNV infection [17]. Local depletion of subcapsular sinus MΦs had little or no effect on either B cell or CTL responses to WNV after it was injected into footpads of mice [18, 19]. However, draining lymph node (LN) MΦs are required for mice to survive a local WNV infection [18]. While specific IFN stimulated gene products have been shown to control WNV replication and spread within MΦs and MOs, relatively little is known about the role of splenic MΦs and MOs in the protection against subcutaneous WNV infection.

Although the spleen is a major site for WNV replication and spread, it is unclear which innate cells in the spleen replicate WNV, control viral dissemination, and/or regulate innate and adaptive immune responses. We decided to test if splenic MΦs were necessary for control of WNV infection. We selectively depleted MΦs from the spleen by injecting mice i.v. with CLL several days prior to infecting them with WNV. This regimen did not affect draining LN MΦs. Mice missing splenic MΦs succumbed to WNV infection after an increased and accelerated spread of virus to the spleen and the brain. WNV-specific Ab and CTL responses were normal in splenic MΦ-depleted mice; however, numbers of NK cells and CD4 and CD8 T cells were significantly increased in the brains of infected mice. Splenic MΦ deficiency led to increased WNV in splenic CD11b- DCs, newly formed MΦs and monocytes. Unlike other splenic myeloid subsets, splenic MΦs express high levels of mRNAs encoding the complement protein C1q, the apoptotic cell clearance protein Mertk, the IL-18 cytokine and caspase 12. Splenic MΦ-deficient mice may be highly susceptible to WNV infection in part to a deficiency in the expression of one or more of these proteins.

## Materials and methods

### Mice

C57BL/6J WT mice were purchased from The Jackson Laboratory (Bar Harbor, ME) and subsequently bred in-house. All mice were housed and maintained in a specific-pathogen-free facility at the University of Washington. B cell-deficient μMT mice were kindly provided by Dr. David Rawlings (Seattle Children’s Research Institute).

### Ethics statement

All animal experiments were performed in strict accordance with the recommendations in the NIH guidelines listed in the Guide for the Care and Use of Laboratory Animals [20], the Animal Welfare Act, and U.S. federal law. All experiments and procedures were approved by and conducted according to the University of Washington Institutional Animal Care and Use Committee (Assurance Number A3464-01, protocol 2242–08). Footpad injections were performed under anesthesia with ketamine hydrochloride and xylazine, and all efforts were made to minimize suffering. The University of Washington Animal Care and Use Program is fully accredited by the Association for Assessment and Accreditation of Laboratory Animal Care International accreditation number 000523, and registered with the USDA, certificate number 91-R-0001.

### Infection with WNV

Female and male 6- to 10-week-old, age- and sex-matched mice were injected under anesthesia in one hind footpad with 10 μL of 100 PFUs of WNV diluted in a vehicle of Hanks buffered salt solution containing 1% FBS. Mice infected with WNV were monitored twice a day for weight, morbidity and mortality. A clinical scoring system was used as follows: 0 = healthy mouse; 1 = ruffled fur, lethargy, hunched posture, no paresis; 2 = very mild to mild paresis; 3 = some paresis in one hind limb or conjunctivitis; 4 = paresis but retains feeling’ possibly limbic; 5 = true paresis; 6 = moribund. Mice with a weight loss of ≥20% of their baseline weight or reaching a score of 5 were euthanized immediately using CO2 followed by cervical dislocation. Mice that were infected with WNV and did not meet the criteria for euthanasia were kept up to 30 days after infection and then sacrificed as described above. A few WNV-infected mice that had not met any criteria for euthanasia at the last check point 12 hours previously (≤5%) were found dead in the cage in spite of twice daily monitoring and added environmental enhancements (gel, moist food and extra nestlet material).

### In vivo depletion of splenic MΦs

WT mice were injected iv with liposomes in 100 μL containing either 0.5 mg CLLs or 0.5 mg control PBS liposomes (PBSL) as described [17]. To verify that splenic MΦs had been depleted, 3 days after CLL or PBSL injection, cell suspensions were prepared from isolated spleens and myeloid subsets quantified as described in the Results.

### Virus and quantification of viral burden

All virus stocks were maintained and characterized within the University of Washington’s Center for Flavivirus Immunity. The WNV infectious clone (WNV-TX) [21,22] was used at 103 PFU/dose, as determined by a plaque assay using baby hamster kidney (BHK) cells. Viral tissue burden quantification was done as described [23]. Briefly, infected mice at various times p.i. were sacrificed and perfused with PBS, and tissues were harvested into centrifuge tubes containing ceramic beads and PBS, then tissues were homogenized once in a homogenizer except brain tissues which went through two cycles of homogenization. Samples were spun down, and the supernatants were analyzed for viral titer with a plaque assay using BHK cells. Viral RNA levels in sera, tissues and sorted cells were determined by quantitative RT-PCR (qPCR) as previously described using Sybr green master mix (Applied Biosystems) and WNV DNA standards (WNV E protein DNA in a plasmid) [11]. Viral RNA was extracted using a QiaAMP viral RNA extraction kit (Qiagen, Valencia, CA), and total RNA was extracted from tissues using RNeasy kits (Qiagen). Sequences for primers used were as previously published [11, 24]: forward primer 5’-TCAGCGATCTCTCCACCA AAG and reverse primer 5’-GGGTCAGCACGTTTGTCATTG. Threshold cycle values from samples analyzed by Sybr green were made relative to the GAPDH housekeeping gene and normalized to WT uninfected samples.

### Quantification of Ab responses

WNV-specific Ab responses were determined as described [23]. Briefly, relative quantities of WNV-specific Abs were measured using a sandwich ELISA and WNV envelope (E) protein purified as described [25]. Serum samples were inactivated for virus by exposure to UV light for 30 min, serially diluted 3-fold starting at 1/20 in 0.1% milk casein in PBS. Goat anti-mouse IgM or goat-anti- mouse IgG sera conjugated to horseradish peroxidase (Southern Biotech, Birmingham, AL) were added at a 1:2,000 or 1:4,000 dilution. A positive reading was determined to be a value above the cutoff value determined from the mean of negative-control wells (naive and/or mock-infected mice) + the standard deviation of the mean X the standard deviation multiplier, f [26]. Negative-control wells contained serial dilutions from at least three individual mice per experiment. To determine neutralizing (nAb) titers, serum samples were analyzed in a plaque reduction neutralization titer (PRNT) assay as previously described] 11, 23].

### Spleen and dLN cell isolation

LNs were harvested suspended in digestion buffer (serum-free RPMI 1640 medium, Thermo Scientific, Waltham, MA) in the presence of 170 μg/mL Liberase (Roche, Pleasanton, CA) and 10 μg/mL DNase I (Roche) and incubated for 25 min at 37°C. LNs were then disrupted mechanically with a needle and syringe and strained through nylon mesh to obtain a single cell suspension. Spleens were injected with 1 mL of digestion buffer then incubated for 30 min at 37°C. Spleens were transferred to a 70um cell strainer, washed with buffer (1xPBS, 2mM EDTA, 0.5% BSA, 10 μg/mL DNase1) to generate a single cell suspension. Cells were then washed with FCS-containing RPMI 1640 medium and red blood cells (RBCs) were lysed with 1X RBC lysis buffer (BioLegend, San Diego, CA) prior to staining for flow cytometry. For isolation of leukocytes from the brain, tissues were harvested and finely chopped with scissors over a wire screen mesh in cold 5% FCS-containing PBS. Cells were then washed with FCS-PBS and resuspended in digestion buffer and incubated for 45 min at 37°C. After adding EDTA to 2mM, tissues were further disrupted by pipetting, passed through strainer into 25% Percoll (Sigma-Aldrich, St. Louis, MO) in RPMI with 1% FCS, 25mM HEPES and spun with brake 20 min at 800xg. The myelin/fat layer was aspirated and cell pellet suspended in PBS without serum. Leukocytes were then suspended in 70% Percoll layer that was underlayed with 30% Percoll, and the cells were spun down for 20 min at room temperature, no brake. Lymphocytes were obtained from the 30%:70% interface and washed with serum-containing RPMI 1640 medium prior to staining for flow cytometry.

### Flow cytometric analyses and identification of cell subsets

At various time points p.i. single cell suspensions of popliteal dLNs, spleen or brain were prepared as above and stained with one or more of the following rat anti-mouse mAbs as described [20]. Biotin anti-F4/80 (eBioscience; clone BM8) with streptavidin- PE or streptavidin PE Cy7 (eBioscience), anti-CD3ε-PerCP Cy5.5 (eBioscience; clone 145-2C11) or anti-CD3ε-PE Cy7 (eBioscience; clone 145- 2C11), anti-CD45R/B220-eFluor450 (eBioscience; clone RA3-6B2) or anti-CD45R/ B220-peridinin chlorophyll protein (CD45R/B220-PerCP) (BD; clone RA3-6B2), anti-CD11c-allophycocyanin (CD11c-APC) (eBioscience; clone N418), anti-NK1.1-PerCP Cy5.5 (eBioscience; clone PK136), anti-CD44-PE Cy7 (eBioscience; clone IM7), anti-CD8α-APC Cy7 (BD; clone 53–6.7), anti-CD4-APC (BD; RM4-5), anti-MARCO (Serotec; clone ED31, anti CD11b (eBioscience; clone M1/70), anti-CD19 (eBioscience; clone MB19-1), anti-Ly6G (BioLegend; clone 1A8), anti-CD45 (eBioscience; clone 104), DAPI (Molecular Probes) All viable-cell events were acquired on an LSR II flow cytometer (Becton, Dickinson, Franklin Lakes, NJ) and analyzed with Flowjo software (Treestar Incorporated, Ashland, OR). To determine the absolute cell numbers of a specific cell population, total cell numbers of harvested tissues were counted and multiplied by the frequency of a specific population (expressed as a percentage of total acquired events).

To detect WNV-specific CD8^+^ T cells, cells were first stained with mAbs to surface markers, washed the cells and then stained them with a 1:100 to 1:250 dilution of MHC class I tetramer-PE at 4°C for 25 min. All samples were stained in PBS containing 2% FCS and 0.05% azide (FACS buffer) at 4°C and fixed with PBS containing 4% paraformaldehyde before acquisition.

For intracellular cytokine staining (ICS), first 4x10^6^ spleen cells from infected mice were first restimulated in vitro with either 1 μM of the CD8^+^ T cell-specific NS4B 9-mer SSVWNATTA peptide [27] or the CD4^+^ T cell-specific NS32066–2080 15-mer RRWCFDGPRTNTILE peptide [28] peptide (Genemed Synthesis Inc., San Antonio, TX) together with GolgiPlug containing brefeldin A (BD Biosciences, San Diego, CA) at 37°C for 5 or 16 h, respectively. Cells were then spun down, washed and stained with mAbs to surface markers, fixed with PBS containing 4% paraformaldehyde, and then permeabilized and stained in FACS buffer containing 0.1% saponin with anti-IFN-γ-Pacific blue (eBioscience; clone XMG1.2), anti-tumor necrosis factor α (TNF-α)-PE (eBioscience; clone MP6-XT22), anti-perforin-PE (eBioscience; clone eBioOMAK-D), or respective isotype control mAbs. The biotinylated WNV epitope-specific peptide MHC class I tetramers were prepared as described [23].

The schemes for the analysis of myeloid subsets in the spleen and dLNs are shown in Fig A in S1 File and Fig B in S1 File, respectively. For analyses of splenocytes, debris, doublets and dead cells were excluded based on side scatter (SSC) and granularity. Myeloid cell populations were defined based on their expression of CD11b and other markers: CD11b^hi^ F4/80^hi^ MΦs; conventional F4/80-CD11c^hi^MHCclassII^hi^ DCs, which were further subdivided into CD11b^-^ DCs and CD11b^+^ DCs; NK1.1^hi^CD11b^+^ CD3^-^ NK cells; NK1.1^hi^CD11b^+^ CD3^+^ NKT cells. NK1.1^-^CD11b^+^CD19/CD3^-^ cells were further subdivided based on SSC and relative Ly6C and Ly6G expression into SSC^lo^ Ly6C^lo^ MOs, SSC^lo^ Ly6C^hi^ MOs, Ly6G^hi^Ly6C^+^SSC^int^ Nphs and SSC^hi^Ly6C^+^Ly6G^+^ eosinophils. CD11b^-^CD3/CD19^+^ cells were subdivided into MHC class II^hi^ B cells and MHC class II^-^ T cells; For analyses of dLN cells, after exclusion of debris, doublets and dead cells, cell populations were defined based on their expression MCH class II and/or CD19/CD3 as B cells (CD3/CD19^+^MHC class II^hi)^ and T cells (CD3/CD19^+^MHC class II^-^); CD3/CD19^-^ cells were further subdivided into NK cells (NK1.1^hi^FSC^lo^), Nphs (CD11b^hi^Ly6G^hi^), CD11b^+^ cDCs, CD11b^-^ cDCs, F4/80^hi^ MΦs and Ly6C^hi^ MOs.

### Sorting of splenic myeloid subsets

Splenocytes prepared as described above were resuspended in sort-buffer (1 x PBS, 2mM EDTA, 0.5% BSA and >10μg/mL DNase1, 10mM HEPES) and incubated with F4/80-biotin α-biotin magnetic beads on ice for 15–20 min. Then cells were washed, resuspended in sort-buffer and added to an LS column attached to the MACS Separator. The columns were washed with 5 mL of sort-buffer and unbound cells were used to obtain other myeloid subsets. The column was removed from the MACS Separator into a new 15 mL conical tube on ice and the RPMs (F4/80 enriched cells) were eluted with 5 mL sort-buffer. Cells obtained from the flow through fraction were incubated with anti-CD11c and anti-CD11b beads, washed, resuspended in sort-buffer and added to a prepared LS column. The LS column was removed and cells eluted with 5 mL sort buffer. Cells were stained with the surface markers for myeloid cell subsets before sorting, except for Streptavidin-BV605 used to stain F4/80 LS enriched cells. For sorting cells from WNV-infected mice, a BSL3 flow cytometry facility with an Aria1 cell sorter in the Department of Immunology’s Cell Analysis Facility Flow Cytometry Core was used. Stained cells were gated to eliminate debris, doublets and dead cells using DAPI. B cells were excluded gating on CD19^-^ cells. RPMs were identified as F4/80^hi^FITC^+^ (autofluorescent) cells. We used the CD11b vs. CD11c markers to separate out CD11c^hi^CD11b^+^ DCs from CD11c^hi^CD11b^-^ DCs. We gated on CD11b^hi^CD11c^-^ cells to isolate Ly6G^+^ neutrophils; then the CD11b^hi^Ly6G^-^ cells were further gated on SSC^lo^F4/80^-^ cells to sort MOs.

### mRNA isolation and qRT-PCR array

Total mRNA was isolated from whole spleen using Qiagen RNeasy minispin columns and using the manufacturer’s protocol. In brief, tissues were homogenized and treated with RLT lysis buffer to obtain lysates for RNA isolation. Isolated RNA was reverse transcribed using a cloned avian myeloblastosis virus reverse transcriptase kit (Invitrogen, Grand Island, NY) to make cDNA. qRT-PCR reactions were performed on ViiA7 RT-PCR instrument (Applied Biosystems, USA) using TaqMan PreAmp Master Mix (ThermoFisher Scientific, USA. Custom TaqMan Array cards (pre-loaded 384-well microfluidic cards) were purchased from ThermoFisher Scientific (list of primers in Table A in S1 File). All reactions were performed according to the manufacturer’s protocols. Gene expression analyses were performed by ΔΔCT method [29] using generated using DataAssist3.1 with 18s and HPRT as endogenous controls as previously described [30]. WNV infected samples were compared to mock-infected samples. PCR-amplification of each cDNA sample was done in triplicates.

### Statistical analyses

All statistical analyses were performed using Prism 6 software (GraphPad Software, USA). Statistical analyses were conducted using Student’s t test with Welch’s correction, 1-way ANOVA or 2-way ANOVA and Tukey’s multiple comparison test. In all figures *P<0.05, **P<0.01, ***P<0.001, ****P<0.0001.

## Results

### Depletion of splenic MΦs by intravenous delivery of clodronate liposomes

**Fig 1:**
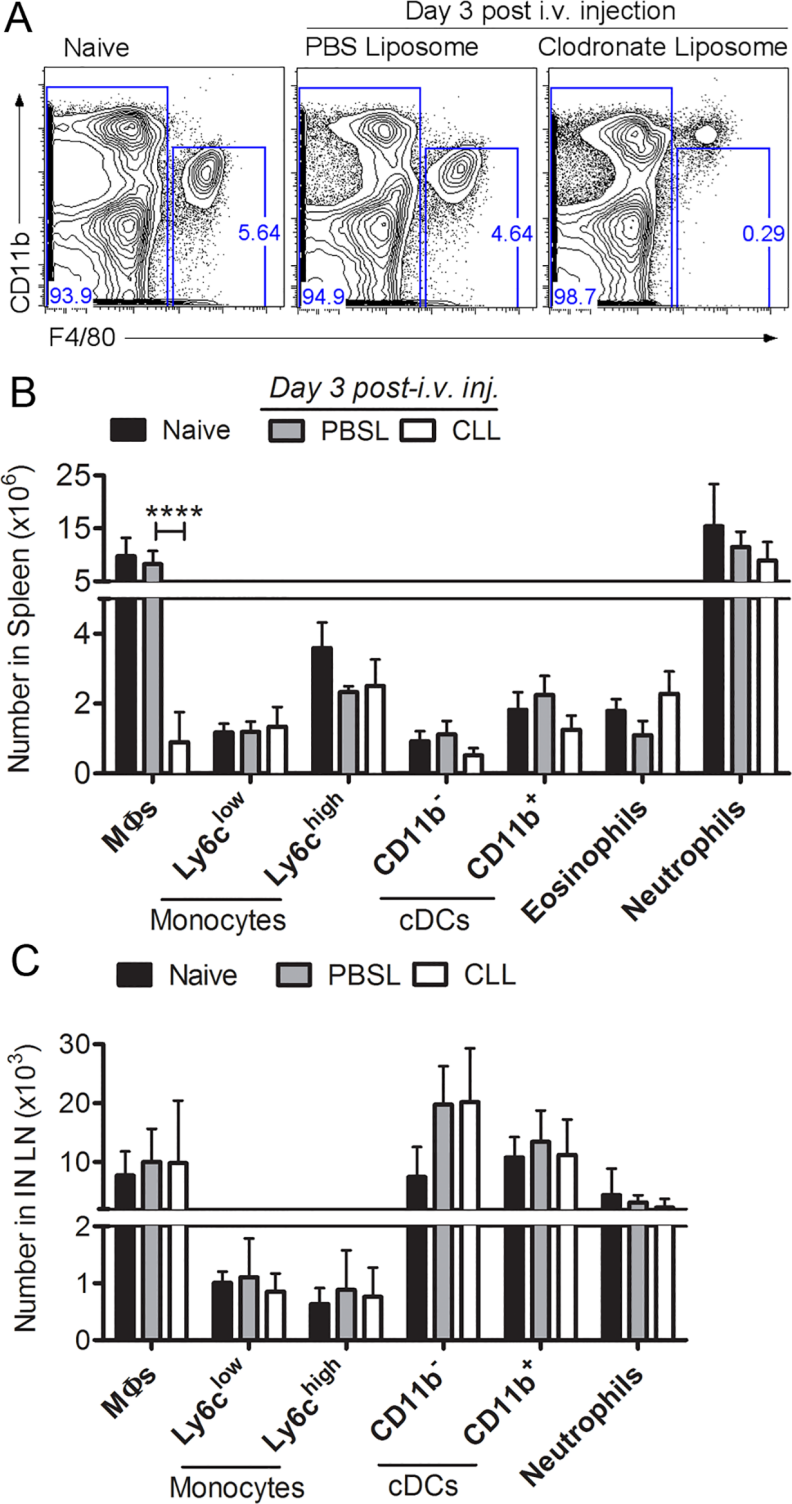
Selective depletion of splenic macrophages by i.v. delivery of clodronate liposomes (CLL). Mice were left untreated (naïve) or injected i.v. with CLL or saline filled liposomes (PBSL) as a negative control; 3 days later spleens were harvested and cells analyzed by multi-color flow cytometry. (A) Representative plots showing CD11b vs. F4/80 staining of splenocytes from naïve and liposome treated mice. (B, C) Numbers for splenic and lymph node populations were calculated based as cell frequency X total spleen cell numbers. Data shown represent one of four separate experiments with similar results. Statistics were performed using 2-way ANOVA and Tukey’s multiple comparison test. P values: * p < 0.05, ** p <0.01, *** p<0.001, **** < 0.0001.

To develop a method for depletion of splenic MΦs, we injected mice i.v. with CLLs or PBS-filled control liposomes (PBSL) and analyzed spleen cell populations 3 days later (**Fig 1**). F4/80^hi^ CD11b^int/low^ splenic MΦs were present in naïve and PBSL-treated control mice but were selectively and significantly depleted in the CLL-treated mice (**Fig 1A and 1B**, gating shown in **Fig A in S1 File**). Other myeloid populations including MOs, DCs, eosinophils and Nphs were not significantly depleted (**Fig 1B**). Splenic MΦs remained depleted 6 days after CLL treatment. The depletion was restricted to MΦs in the spleen, as MΦs were not depleted from inguinal draining LNs (dLNs) **(Fig 1C**, gating shown in **Fig A in S1 File**) or popliteal dLNs. In addition, NK, NKT cells, T cells, and B cells were not depleted from the spleen or dLNs after CLL treatment (**Fig A in S1 File**). Thus, i.v. delivery of CLL resulted in selective and sustained depletion of splenic MΦs.

### Mice depleted of splenic MΦs are highly susceptible to WNV

**Fig 2:**
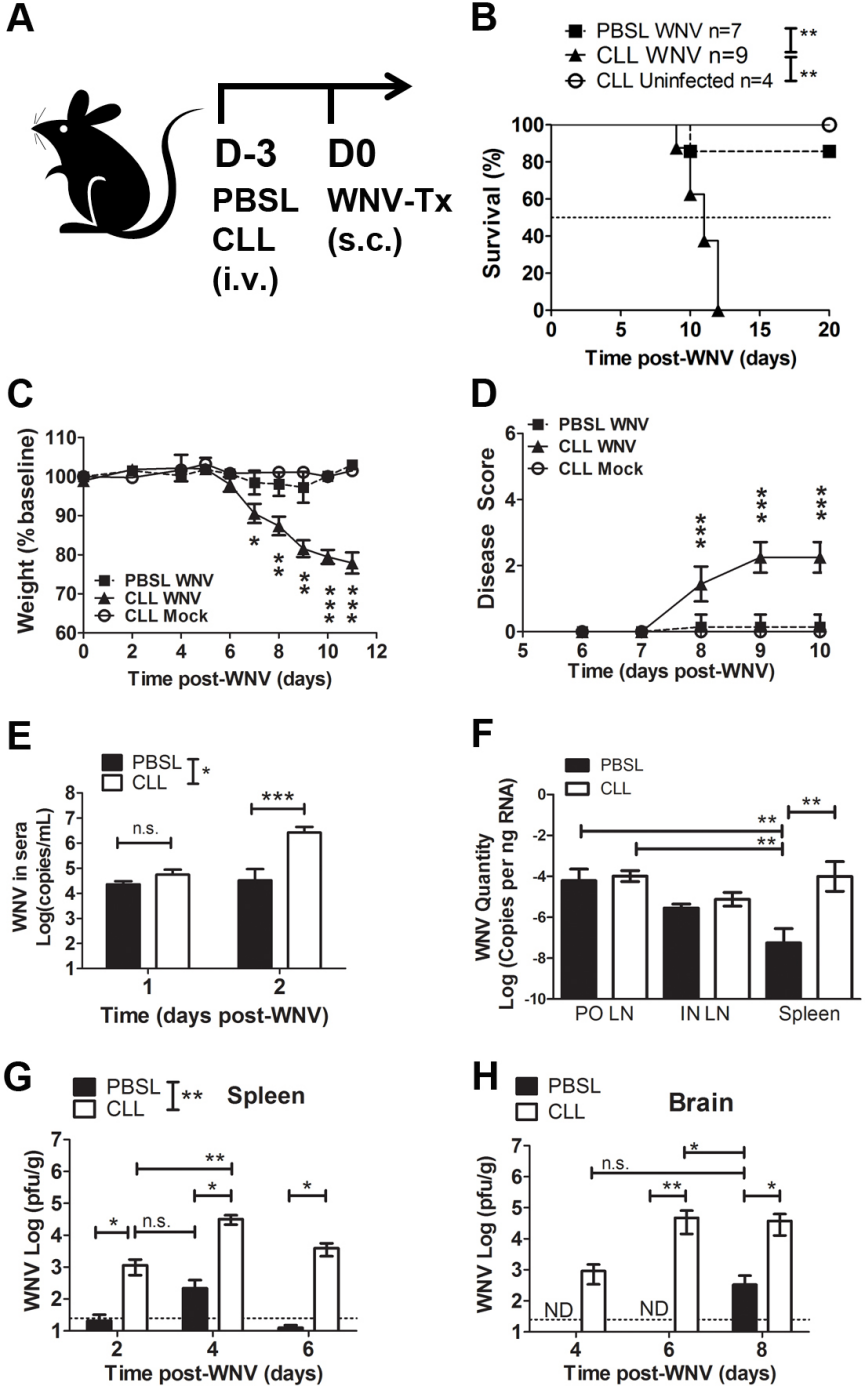
Splenic MΦs are necessary for control of WNV infection. **(A)** Experimental design; mice were treated with CLL or PBSL 3 days prior to subcutaneous (s.c.) footpad inoculation with WNV (1000 PFU) or vehicle control (CLL Mock). Mice depleted of splenic MΦs (triangles), unlike infected PBSL controls (squares) or CLL Mock mice (circles) all succumbed to WNV infection (MST = 11 days) **(B)**; CLL treated mice had significantly decreased body weight at days 7–10 p.i (**C**) and had significantly increased disease scores at day 8–10 p.i. (**D**). (**E**) WNV RNA in sera (qPCR) of CLL-treated (white) vs. PBSL-treated control (black) mice at day 1 and day 2 post-WNV infection. (**F**) WNV RNA in popliteal (PO) and inguinal (IN) dLNs and in spleen of CLL treated (open) vs. PBSL control (black) mice at day 2 post-WNV infection. Viral levels determined by plaque assay in spleens (**G**) and brains (**H**) of CLL-treated (open) and PBSL-treated (black) mice days 2–8 pi. Statistics used were **B**: Log-Rank Test; **C,D**: Student’s t tests; and **E-H**: 1-way Anova plus Tukey’s post-test. P values: * p < 0.05, ** p <0.01, *** p<0.001, ND = not detected.

To determine whether splenic MΦs are required for protection against WNV infection, we compared the susceptibility of mice treated with either CLL or PBSL 3 days prior to footpad inoculation of WNV-TX (**Fig 2A**). All mice depleted of splenic MΦs prior to WNV infection succumbed to infection by day 12 post-infection (median survival time, 11 days), compared to 14% of infected control mice (p = 0.0015) (**Fig 2B**). Splenic MΦ-depleted mice began to show weight loss and clinical symptoms within 7–8 days p.i. (**Fig 2C and 2D**) and also had increased levels of WNV in the blood as early as day 2 p.i. (**Fig 2E**). These data demonstrate that splenic MΦs are required for survival after WNV infection and suggest that they might be essential to control virus replication in the spleen very early after WNV infection.

### Control of WNV replication in the spleen and CNS requires splenic MΦs

To further examine the role of splenic MΦs during WNV infection, we determined the levels of WNV RNA at the WNV dLN inoculation site and in the spleen at 2 days p.i.; although PBSL-control- and CLL-treated mice had similar levels of WNV RNA in popliteal and inguinal dLNs, CLL-treated mice had higher levels of WNV RNA in the spleen at day 2 post-WNV infection (**Fig 2F**). WNV replication in the spleens of WT mice typically peaks about day 4 p.i., and then decreases and is cleared within 6 to 8 p.i.; this course of infection is evident even in innate immune defective MAVS^-/-^ mice [7]. However, splenic MΦ-depleted mice had both a higher frequency of detectable WNV (67% positive versus 17% positive in PBSL controls) and higher infectious virus levels in the spleen even at 2 days p.i. (**Fig 2G**). Splenic MΦ-depleted mice all had detectable virus in the spleen by day 4 p.i., compared to 50% of the PBSL-treated mice, and greater than a hundred fold increase in viral load (p = 0.002), and, unlike the control mice, continued to have high levels of virus at day 6 p.i. However, at day 4 p.i. the splenic MΦ-depleted and control mice did not differ in their ability to control virus in the kidneys and liver.

The lack of splenic MΦs also led to an increased viral burden in the brain. Infectious virus was detectable in the brains as early as day 4 p.i. in splenic MΦ-depleted mice, whereas control mice had no detectable virus at this time (**Fig 2H**). As the infection progressed, WNV levels increased and 80% of splenic MΦ-depleted mice by day 8 p.i. had detectable WNV in the CNS. Together, these data show that splenic MΦs are required early during infection for control of virus replication in the spleen and that in their absence, virus rapidly spreads to the brain.

### WNV-specific Ab responses are elevated in splenic MΦ-depleted mice

**Fig 3:**
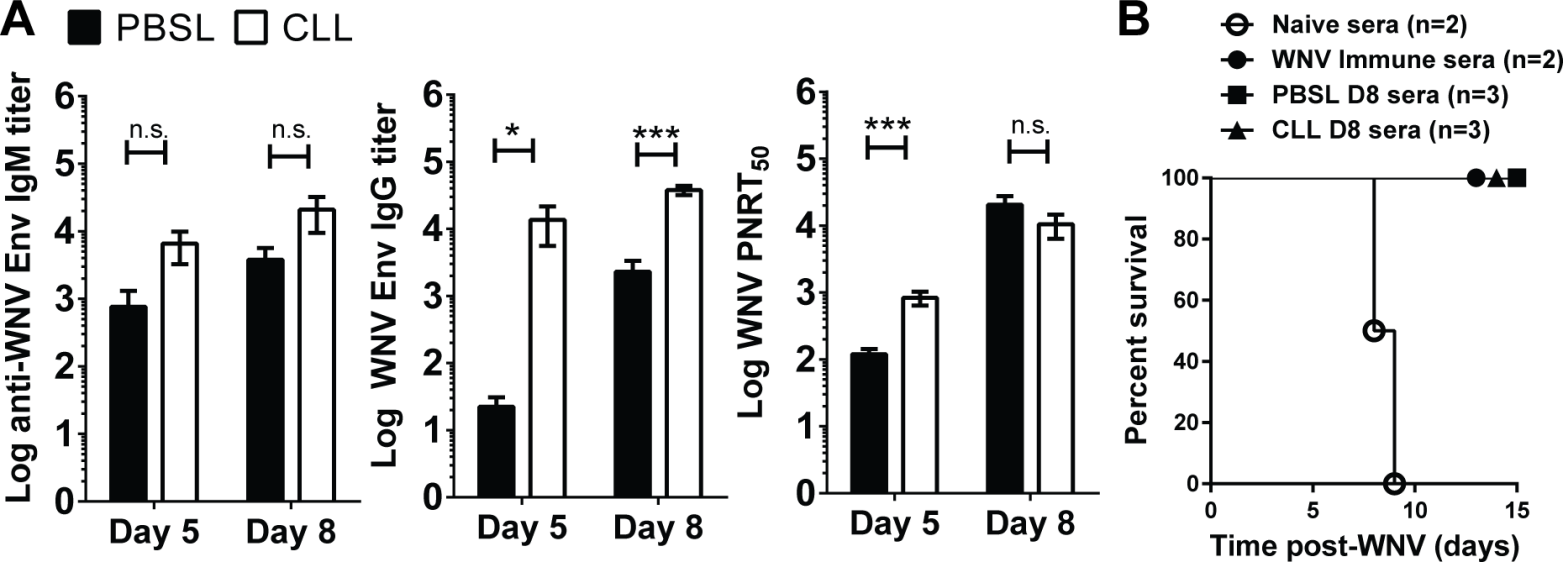
WNV-specific Ab responses in splenic MΦ-depleted mice. Mice were treated as in Fig 2A with CLL (open) or PBSL (black), and sera were collected at days 5 and 8 p.i. (**A**) Anti-WNV Env IgM and IgG titers were determined by OD values that were 3 standard deviations above the mock controls. PNRT_50_ represents the reciprocal dilution at which 50% of plaques were neutralized, The combination of two independent experiments is shown. (**B**) Serum samples were obtained from naïve, WNV immune (>15 days post-WNV), and at day 8 post-WNV from PBSL or CLL treated mice. Heat inactivated pooled sera were then injected into μMT B cell-deficient mice 1 day prior to and 1 day after inoculation of mice with 100 PFU WNV. A pool of sera from naïve mice was used a negative control and provided no protection. Two-tailed Student's t test, * p < 0.05, ** p <0.01, *** p<0.001.

WNV-specific Abs are important for controlling virus early after infection [31,32], and MΦs can contribute to the development of effective humoral responses [15,33,34]. Therefore, we tested whether the lack of splenic MΦs might hinder the development of WNV specific Ab responses, which in turn might account for the increased WNV in the spleen and CNS in splenic MΦ-depleted mice, as occurs in B cell deficient (μMT) mice [31]. We examined sera from infected CLL- and PBSL-treated mice for IgM and IgG responses to recombinant WNV envelope (Env) protein (**Fig 3A**). Surprisingly, splenic MΦ-depleted mice, rather than having reduced levels of WNV-specific Ab or nAb, had increased WNV specific IgG and nAb compared to controls. Both Env-specific IgG at days 5 and 8 p.i. and nAb at day 5 p.i. were significantly higher in splenic MΦ-depleted mice compared to controls. These data suggest that the increased levels of Ab in CLL-treated mice were not sufficient to clear the increased WNV in these mice.

To directly assess the quality of the Abs produced in WNV-infected splenic MΦ-depleted mice, we compared sera obtained from splenic MΦ-depleted mice to sera from PBSL-treated mice or immune sera from WNV-infected WT controls for their ability to protect otherwise susceptible B cell deficient (μMT) mice (**Fig 3B**) [32]. A pool of sera obtained from WNV-infected CLL-treated mice at day 8 p.i. was as effective as a pool of sera from infected control mice in protecting μMT mice from dying. Also, after WNV infection B cell numbers in the spleen were similar between splenic MΦ-depleted mice and controls at day 8 p.i., although there was a small decrease in numbers of PNA^+^ germinal center (GC) B cells in splenic MΦ-depleted mice (**Fig B in S1 File)**. Thus, splenic MΦ-depleted mice that succumb to WNV infection do produce Abs that can protect.

### WNV-infected splenic MΦ-depleted mice generate WNV-specific T cells that enter the CNS but fail to control WNV replication

**Fig 4:**
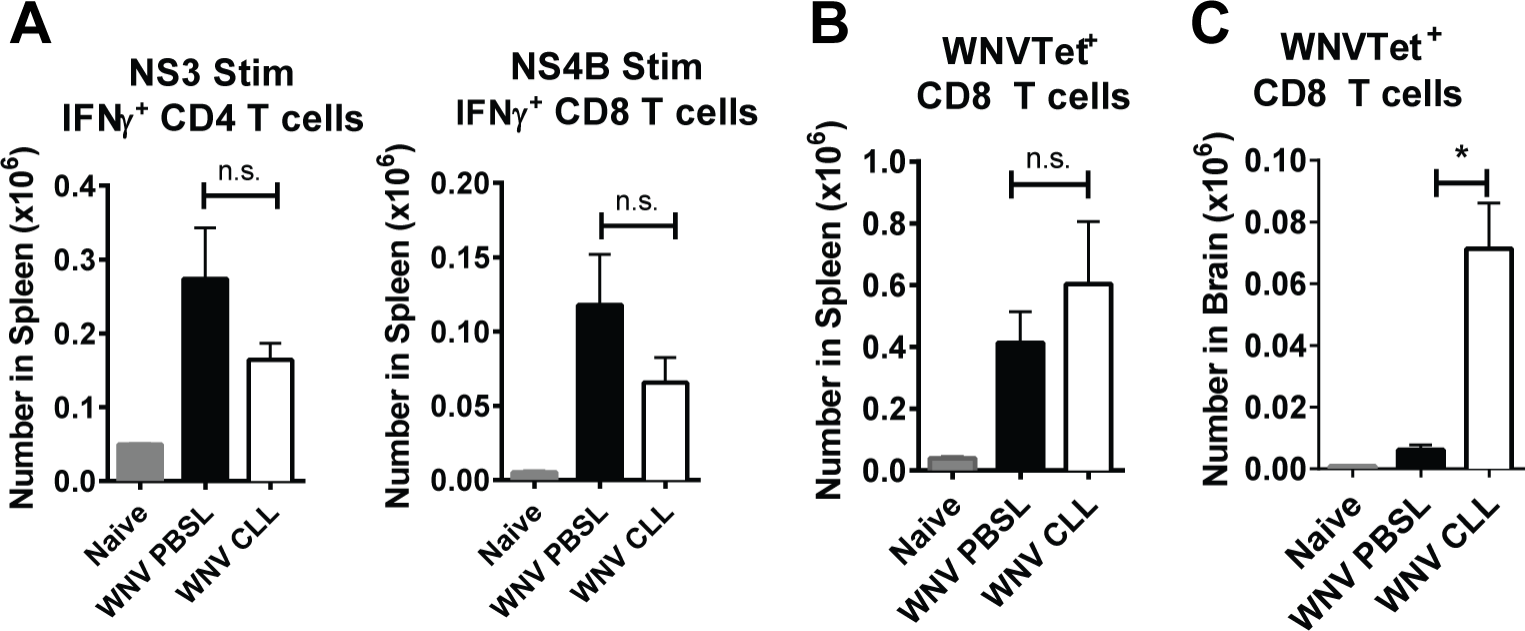
Generation of WNV-specific CD4 and CD8 T cells in WNV-infected splenic MΦ-depleted mice. Mice were treated as in Fig 2A with CLL (open) or PBSL (black) and spleens and brains were harvested at day 8 p.i. (**A**) Splenocytes were stimulated with an NS3 peptide or an NS4b peptide and then analyzed for IFNγ expression within CD4 and CD8 T cells, respectively. (B) Splenocytes were stained with NS4b tetramers to enumerate WNV-specific (WNVTet+) CD8 T cells (C) Leukocytes from brains were stained with NS4b tetramers to enumerate WNVTet+ CD8 T cells. Statistics are shown for 1-way Anova plus Tukey’s post-test. P values: * p < 0.05, ** p <0.01, *** p<0.001.

**Fig 5:**
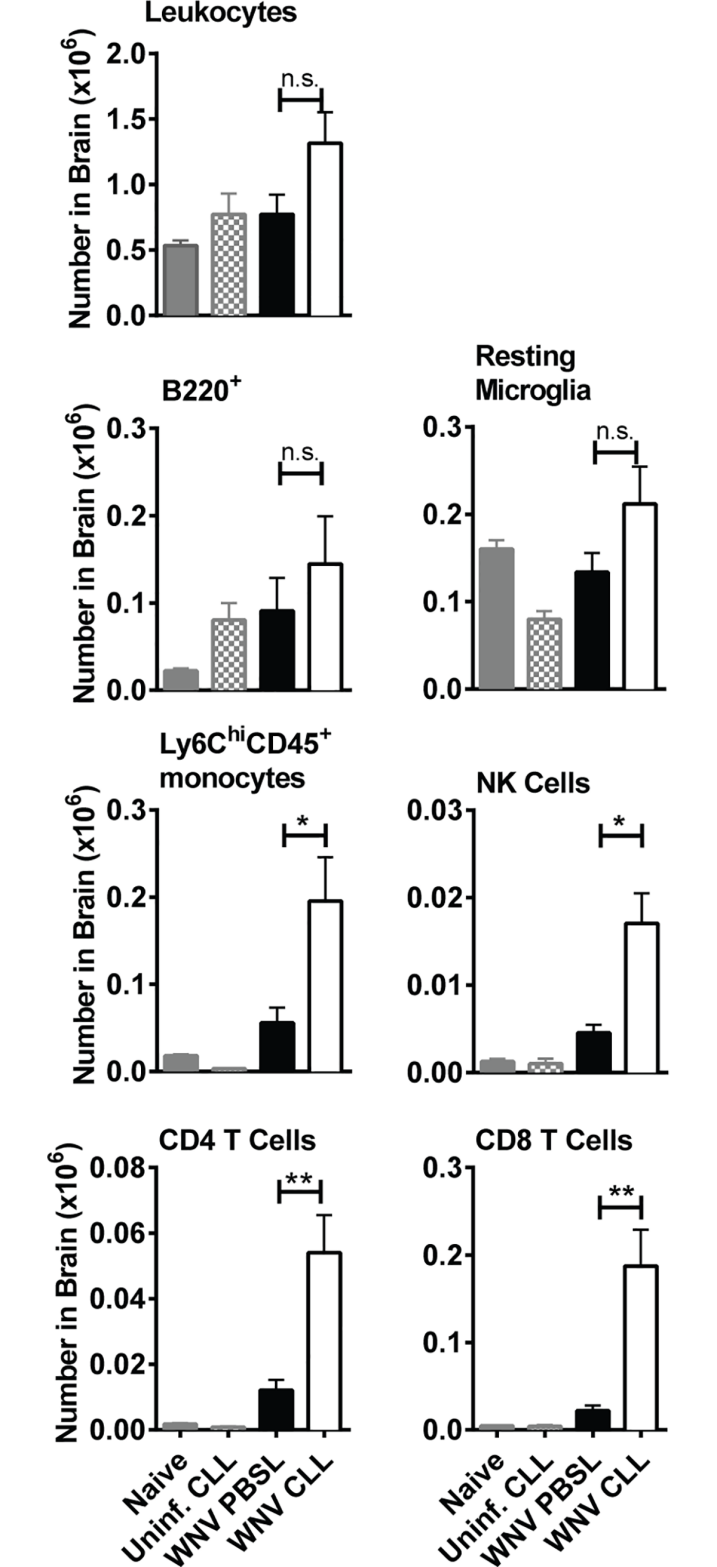
Cellular infiltration into the brains of WNV-infected splenic MΦ-depleted mice. Brains were isolated from mice treated as in Fig 2A with either CLL (open) or PBSL (black), or from uninfected naïve mice (grey) or CLL treated mice (grey checked bar). Brains were harvested at day 8 post-WNV infection. Cell suspensions were stained with mAbs to surface markers and subsets quantified using flow cytometry as: CD45^+^ leukocytes, B220^+^ B cells, CD45^lo^ CD11b^+^ Ly6C^-^ Ly6G^-^ resting microglial cells (52), CD45^+^ Ly6C^hi^ CD11b^+^ Ly6G^-^ monocytes, CD45^+^ NK1.1^+^ NK cells, CD45^+^ CD3^+^ CD4^+^ CD4 T cells, and CD45^+^ CD3^+^ CD8^+^ CD8 T cells. The results shown are the combined result of two independent experiments with similar results. Statistics shown are for two-tailed Student's t test, * p < 0.05, ** p <0.01, *** p<0.001.

Since both CD4 and CD8 T cells are required for protective immunity against WNV [7,10], we assessed if T cell responses were compromised after WNV infection of splenic MΦ-depleted mice. We measured WNV-specific CD4 and CD8 T cell responses (**Fig 4**). Splenocytes were isolated from either untreated naïve mice or from CLL- or PBSL-treated mice day 8 p.i. and cultured in vitro with immunodominant peptides from either WNV NS3 protein or NS4B protein, which stimulate WNV-specific CD4 T cells or CD8 T cells, respectively (**Fig 4A and 4B**). CLL-treated mice had somewhat lower numbers of total CD4 and CD8 T cells than control mice in the spleen (see **Fig 5B**); however, levels of WNV-specific IFN-γ-producing CD4 and CD8 T cells were not significantly different between groups (**Fig 4A**). Likewise, NS4B tetramer-binding CD8 T cells in the spleen were not different between splenic MΦ-depleted mice and PBSL-treated controls (**Fig 4B**). In contrast, NS4B tetramer–binding CD8 T cell levels within the brain were significantly increased in splenic MΦ-depleted mice 8 days p.i. (p = 0.01) (**Fig 4C**). Together these data show that WNV-specific T cell responses were generated in the splenic MΦ-depleted mice and that a greater number of WNV specific CD8 T cells trafficked to the brain in splenic MΦ-depleted mice compared to controls. The brains of the splenic MΦ-depleted mice had increased levels of virus (**Fig 2H**) in spite of the significant recruitment of WNV-specific CD8 T cells.

### Increased cellular infiltration in the brains of WNV-infected splenic MΦ-depleted mice

We next compared the number of leukocyte subsets in the brains of WNV-infected mice pretreated with either CLL or PBSL. Total numbers of leukocytes, B220^+^ B cells and microglia cells in the brains of splenic MΦ-depleted mice were not significantly different from control mice. However, numbers of Ly6C^hi^ MOs, NK cells, CD4 T cells and CD8 T cells were significantly increased at day 8 pi (**Fig 5**). Thus, the high level of WNV in the brains of splenic MΦ-depleted mice (**Fig 2H**) is not simply due to lack of immune cells; rather the numbers of potential effector cells were increased. Interestingly, at the same time point a different pattern was evident in the spleens of MΦ-depleted mice: the cell populations elevated in the brain—Ly6C^+^ monocytes, NK cells, CD4 T cells and CD8 T cells- were instead decreased in the spleen (**Fig B in S1 File**). F4/80^hi^ MΦs were present in the spleens of MΦ-depleted mice at day 8 p.i. (11 days after CLL treatment) but remained significantly lower than MΦ levels in control mice (**Fig B in S1 File**). Together these data suggest that the decreased numbers Ly6C^+^ MOs and other subsets in the spleen may be due to increased trafficking of these same populations to the brain.

### Splenic MΦ deficiency leads to increased WNV expression in splenic CD11c^+^ CD11b^-^ DCs, newly formed MΦs and monocytes

**Fig 6:**
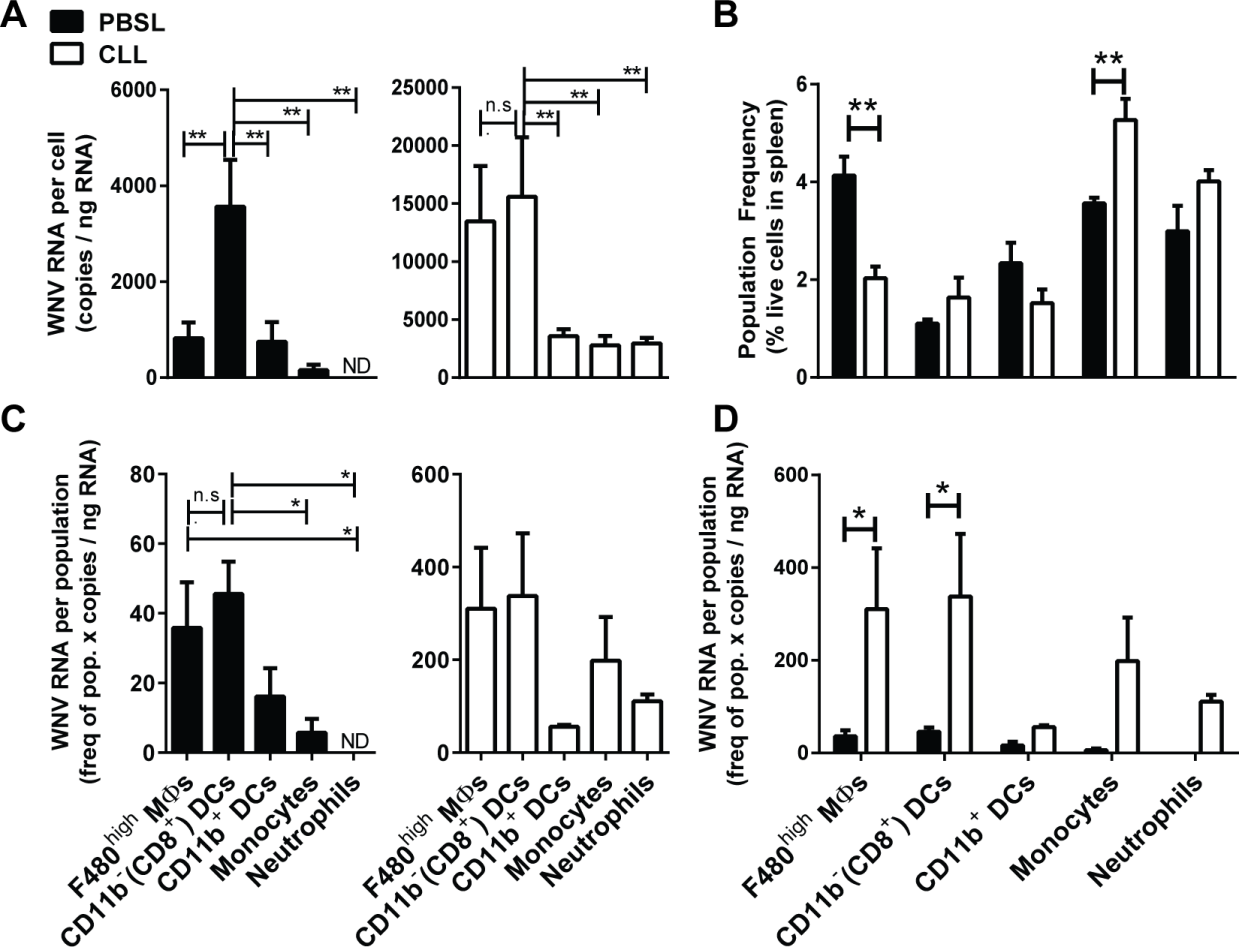
Quantification of WNV viral RNA in myeloid cells from WNV infected mice. Mice were treated with CLL (open bars) or PBSL (black bars) 3 days prior to footpad inoculation with WNV (1000 PFU); spleens were harvested at day 3–4 p.i., and splenocytes were enriched using magnetic beads for CD11b^-^, CD11c^-^, or F4/80^hi^ cells (see Methods). Purified myeloid populations were obtained by cell sorting and RNA isolated for quantization via qPCR. (**A**) WNV quantities/cell indicate that in infected PBSL control mice CD11b^-^ DCs had significantly higher WNV RNA levels than other myeloid subsets. 1-way ANOVA (B) Population frequency determined by FACS analysis on total spleen single cell preparations. 2-way ANOVA with Bonferroni post-test (C) WNV quantity per population indicates that splenic F4/80^hi^ MΦs and CD11b- DCs had similar levels of WNV, while CD11b^+^ DCs and MOs had much less. 1-way ANOVA. (D) CD11b^-^ DCs and repopulating F4/80^hi^ MΦs had significantly higher WNV RNA levels per population in CLL treated mice vs. control mice. 2-way ANOVA with Bonferroni post test. Data represent the combined data from 3 independent experiments. * p < 0.05, ** p <0.01.

To compare expression of WNV in splenic myeloid populations obtained from WNV-infected splenic MΦ-depleted mice to infected control mice, we enriched splenic myeloid populations from WNV-infected mice and examined in detail WNV expression in five myeloid subsets isolated by cell sorting (see Materials and Methods). Little or no WNV RNA was found in splenic B cells and other non-myeloid splenic cells. We extracted RNA from each purified myeloid subset and quantified WNV RNA expression on either a per cell basis (**Fig 6A**) or on a per population basis (**Fig 6C**) by qPCR. In PBSL-treated control mice infected 3–4 days previously, WNV RNA was not detectable in splenic Nphs, was low in splenic MOs and was expressed a little higher in CD11c^+^CD11b^+^ DCs and F4/80^hi^ MΦs. However, it was expressed at high levels in CD11c^+^CD11b^-^ DCs (**Fig 6A**). These data demonstrate that splenic CD11c^+^CD11b^-^ DCs, which are mainly located in T cell zones and active in cross-presentation of antigens [35], are a major target for WNV infection.

During infection of splenic MΦ-depleted mice, the same pattern of WNV expression was evident (**Fig 6**, white bars), except that overall levels of viral RNA were significantly increased (2-way ANOVA p = 0.002). CD11c^+^CD11b^-^ DCs continued to express more WNV than CD11c^+^CD11b^+^ DCs. However, after splenic MΦ depletion, significant WNV RNA was now evident in both MOs and Nphs. Thus, when mature F4/80^hi^ MΦs are depleted, WNV control is altered, making some myeloid cells more susceptible to WNV. Furthermore, F4/80^hi^ MΦs, which had begun to repopulate the spleen 7 days after initial MΦ depletion, had similar levels of WNV RNA on a per cell basis as the CD11b^-^ DCs, whereas in control mice, the mature splenic MΦs had much lower level of WNV RNA than CD11b^-^ DCs (**Fig 6A**). These same patterns of WNV expression were evident when we calculated the total amount of WNV RNA contributed by each splenic cell population by multiplying the frequency of each splenic myeloid subset (**Fig 6B**) by the amount of WNV RNA/cell each subset produced (**Fig 6A**). The CD11c^+^CD11b^-^ DC and F4/80^hi^ MΦ populations contributed the most WNV RNA in both control and CLL-treated mice and at similar levels (**Fig 6C and 6D**). The MOs and Nphs also contributed significant amounts of WNV but only in the CLL-treated mice. Overall the infected splenic MOs in splenic MΦ-depleted mice had 17.5 times more WNV RNA than in controls (Student’s t test p = 0.02). These infected MOs may be a source of MOs infiltrating and bringing WNV to the brains of splenic MΦ-depleted mice (**Fig 5**). The fact that mature F4/80^hi^ MΦs in control mice and repopulating F4/80^hi^ MΦs in CLL-treated mice differed in their level of WNV expression suggested that the MΦs repopulating the spleen might be phenotypically distinct from resident MΦs. Indeed, the newly repopulating F4/80^hi^ MΦs from CLL-treated mice compared to resident MΦs from PBSL-treated mice had significantly increased size and granularity and higher levels of CD11b.

### Gene expression in splenic F4/80^hi^ MΦs from naïve uninfected or WNV-infected mice

**Fig 7:**
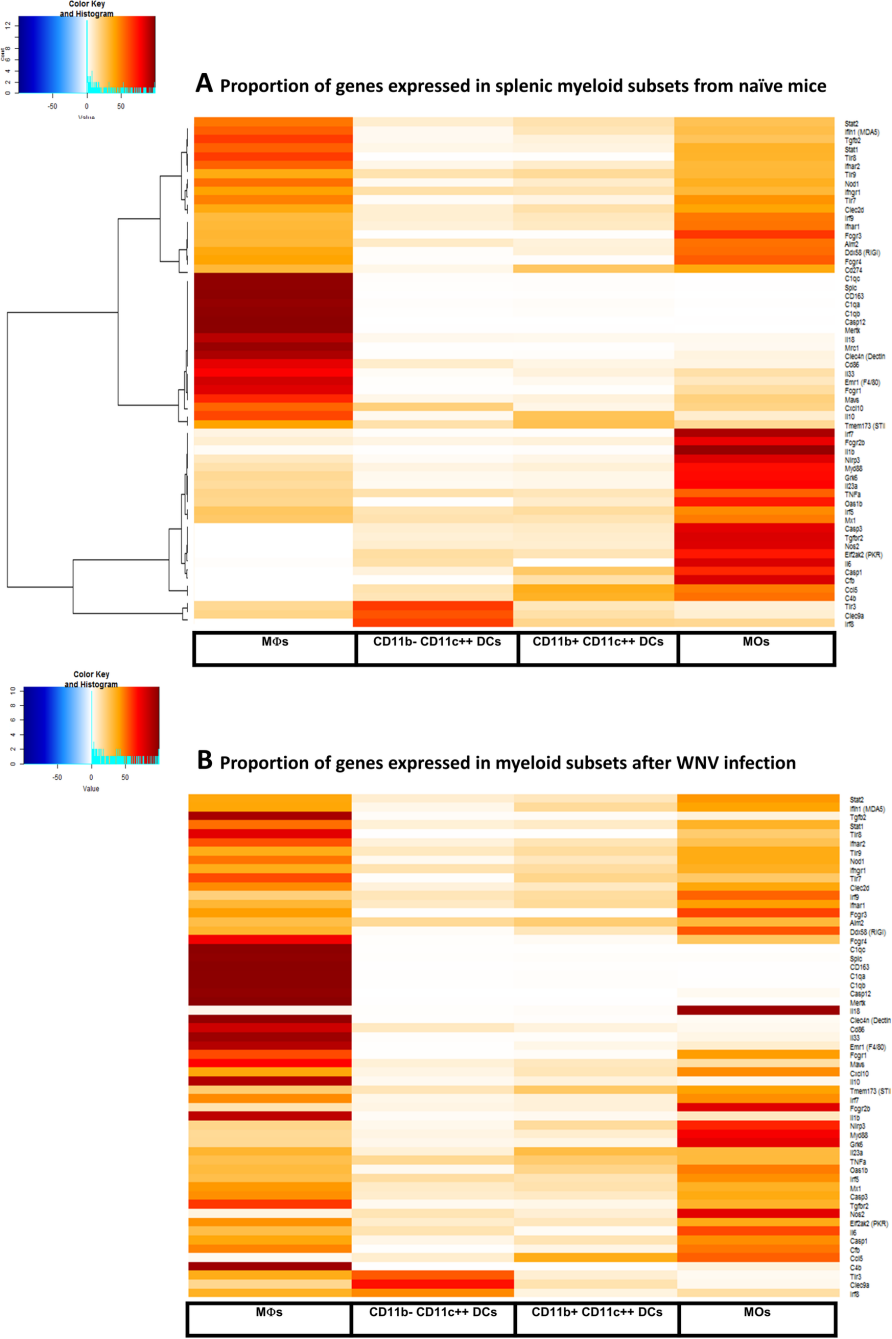
Expression of immune-associated genes in splenic myeloid subsets before and after WNV-infection. F4/80^hi^ MΦs, CD11b^-^ DCs, CD11b^+^ DCs or CD11b^+^SSC^lo^ MOs splenic subsets were isolated by cell sorting from either naïve B6 mice or from PBSL-treated mice infected 4 days previously with 1000 pfu WNV/Tx. RNA was isolated, after which expression of 108 immune-associated genes were determined (see Materials and Methods and Table A in S1 File). Heat maps show the gene expression/population for the 4 subsets relative to the total gene expression in myeloid cells obtained from (**A**) naïve mice (mean of 2 independent experiments) or (**B**) WNV-infected mice (mean of 3 experiments). Ratios were loaded into R statistical programming language (version 3.2.0). Pearson correlation was performed on ratios and displayed in R/Bioconductor using the gplots package. Gene expression/population shown is calculated as expression of the given population X the frequency of the same population in the spleen. Details of expression levels are available in Tables B and C in S1 File.

To assess antiviral genes that may be deficient in splenic MΦ-depleted mice, we used microfluidic qPCR arrays to analyze gene expression in splenic myeloid populations isolated by cell sorting from naïve mice or PBSL-treated mice 4 days after WNV infection. We compared expression of 108 immune-associated genes (**Table A in S1 File**) in F4/80^hi^ MΦs, CD11c^+^CD11b^-^ DCs, CD11c^+^CD11b^+^ DCs or CD11b^+^SSC^lo^ MOs (gating used for cell sorting in **Fig A** in S1 File) and calculated the relative contribution of a myeloid subset to the gene’s expression by multiplying the gene expression levels times the frequency of the splenic population. A number of genes were highly expressed in F4/80^hi^ MΦs isolated from uninfected mice and were significantly much higher compared to the expression in other subsets (**Fig 7A**, **Table B in S1 File**). Thus, the myeloid cells remaining in splenic MΦ-depleted mice are deficient in these genes. Some of these genes, as expected, encoded known MΦ-associated markers like CD163 and Clec4n (Dectin-2). Several genes known to be important for control of WNV infection such as C1q genes were selectively expressed in splenic MΦs. Both C4^-/-^ and C1q^-/-^ mice succumb to WNV infection [36]. Other genes expressed at high levels in splenic MΦs have not yet been directly implicated in the control of WNV but function either in innate immune (e.g., *TLR8*, *IL18*, *Clec4n* and *Casp12*) or adaptive immune responses (e.g., *Tgfb2* and CD86).

CD11b^-^ DCs, unlike the other myeloid subsets we examined, contained high levels of WNV 4 days after infection of control mice (**Fig 6A**). This suggested that the CD11b^-^ DCs might have reduced constitutive levels of genes known to play a role in limiting WNV infection. Indeed, compared to MΦs, MOs and even CD11b^+^ DCs, the CD11b^-^ DCs from naïve mice express much lower levels of RIG-I, MDA5 and Mavs (**Fig 7A, Table B in S1 File**), which are known to play important roles in resisting WNV infection [7,8].

We also evaluated gene expression in these same splenic myeloid subsets, isolated 4 days after WNV infection of PBSL-treated control mice. The expression of a set of genes that increased about 2.5 fold more compared to expression in myeloid cells from uninfected mice is shown in **Fig 7B** and **Table C in S1 File**. These genes fell roughly into three groups. The first group was expressed only or mainly in splenic MΦs and included, as expected, genes highly expressed in splenic MΦs from uninfected mice such as Mertk, C1q proteins and IL18. Notably, C4b was dramatically and almost exclusively upregulated in MΦs from infected mice. The genes encoding the regulatory cytokines IL-10 and IL-33 were also expressed principally in MΦs before infection and upregulated after infection. A second group of genes that were upregulated after WNV infection were expressed in splenic MΦs but not exclusively. Some of the genes expressed in MΦs were also expressed in MOs but with little or no expression in the DC subsets, e.g., those encoding FcRγl, Cfb, IRF7 and TGFßR2. These data suggest that in the presence of splenic MΦ deficiency, splenic MOs in particular may provide proteins that otherwise would be deficient in mice missing splenic MΦs. A third group of genes were increased after WNV infection but expressed mainly in non-MΦs. Notably, Nos2 was strongly induced after WNV infection but mainly in splenic MOs.

### Expression of genes in splenic F4/80^hi^ MΦs that function to sense WNV RNA

**Fig 8:**
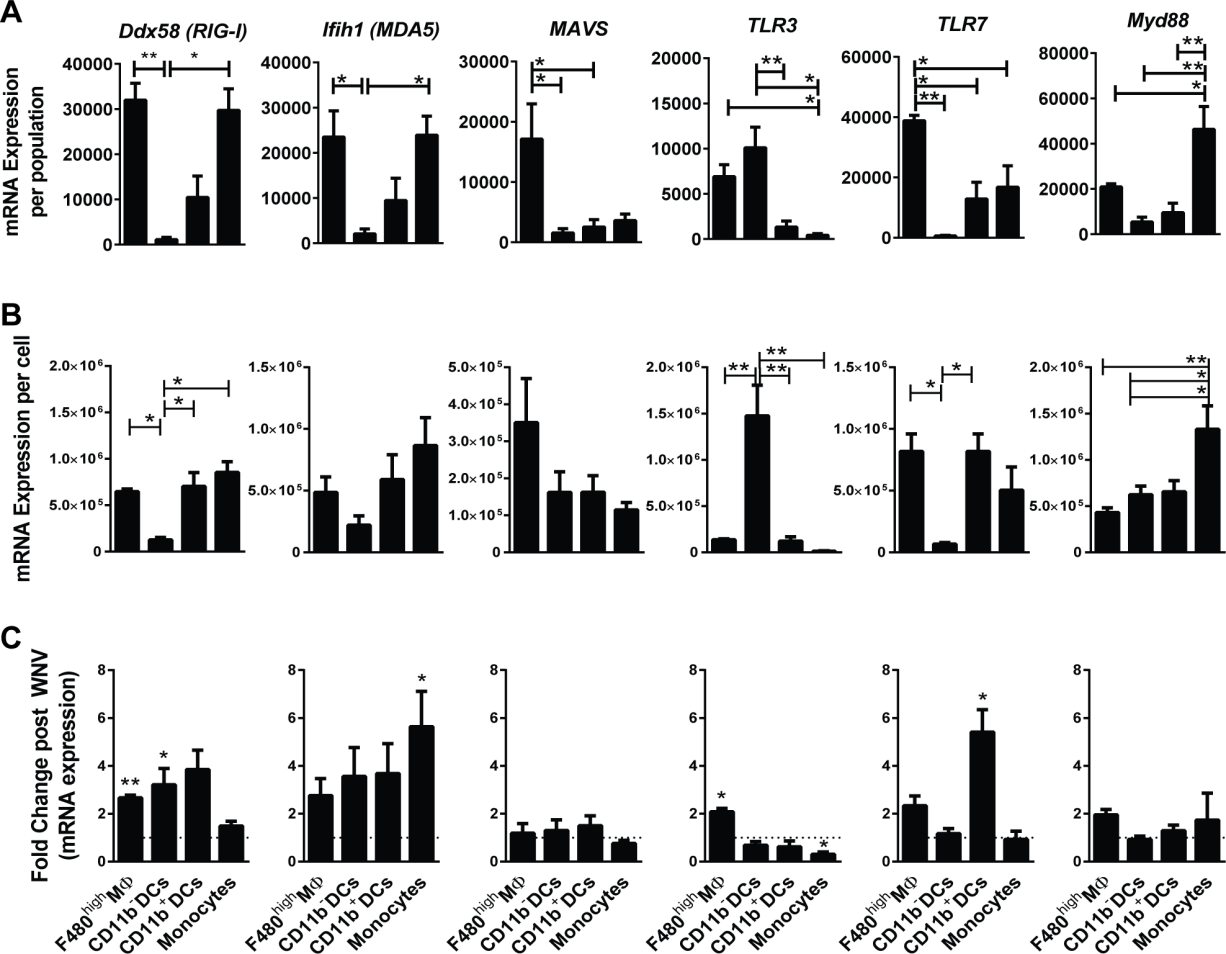
Differential expression in splenic myeloid cell subsets of genes encoding RNA sensing and signaling proteins. Control (PBSL) mice were infected with WNV and spleens were harvested 4 days post-WNV. Myeloid populations (F480^high^ MΦs, CD11b^-^ DCs, CD11b^+^ DCs, and MOs) were isolated by FACS, RNA extracted, and gene expression quantified by qPCR array cards. Gene expression per population shown is calculated as gene expression of the given population X the frequency of the same population in the spleen (expression per cell). (**A)** F480^high^ MΦs express high levels of mRNAs encoding the cytosolic sensors Ddx58 (RIG-I) and Ifih1 (MDA5) as well a large proportion compared to other subsets of the mRNA encoding downstream adapter MAVS. (**B)** F480^high^ MΦs, CD11b^+^ DCs and MOs have high levels of mRNA for *Ddx58* (RIG-I) and *Tlr7* compared to CD11b^-^ DCs, which express more *Tlr3***. (C)** Fold change was determined by dividing expression in myeloid populations from WNV-infected mice by that of the same populations from naïve controls. Splenic myeloid cells from WNV infected control mice increased mRNA expression of some RNA sensors, but not for the downstream adapters *MAVS* and *Myd88*. For all plots, statistics shown are from 1-way ANOVA *p < 0.05, ** p < 0.01. Data represent the combined data from three independent sort experiments.

To assess further how splenic myeloid populations may sense WNV RNA, we compared in more detail the expression in F4/80^hi^ MΦs, CD11b^-^ DCs, CD11b^+^ DCs, and MOs obtained from WNV-infected mice of genes involved in sensing viral RNA and programming type I IFN responses. (**Fig 8**). F4/80^hi^ MΦs and monocytes, unlike DCs, were the major myeloid sources of basal expression of the genes encoding the viral sensors RIG-I (Ddx58) and MDA5 (Ifih1), and F4/80^hi^ MΦs expressed more than 2/3 of the genes encoding the adaptor downstream of RIG-I and MDA5, MAVS (**Fig 8A**). Expression of *MAVS* on a per cell basis was also higher in F4/80^hi^ MΦs than in DC subsets (**Fig 8B**). And as noted above, CD11b^-^ DCs expressed very low mRNA levels of these sensors. A comparison of expression after WNV infection to expression in subsets from uninfected mice (**Fig 8C**) revealed that as, expected, both *Ddx58* and *Ifih1* expression increased to varying degrees in several myeloid subsets after infection, but *MAVS* gene expression did not. And even after infection, the CD11b^-^ DCs expressed relatively low amounts of mRNAs encoding the RIG-I and MDA5 sensors.

A different pattern was evident for the genes encoding the RNA sensors TLR3 and TLR7 and the downstream adaptor, Myd88: monocytes contributed most of the *Myd88* and expressed significantly higher levels per cell of *Myd88* (**Fig 8A and 8B**). Like *MAVS*, *Myd88* expression did not change significantly in splenic myeloid subsets after WNV infection (**Fig 8C**). As with the components upstream of MAVS, genes encoding TLR3 and TLR7 upstream of MyD88 increased after WNV infection: *TLR3*, while constitutively expressed at high levels in CD11b^-^ DCs, was increased in F4/80^hi^ MΦs after infection; however, on a per cell basis, most of the *TLR3* was found in CD11b^-^ DCs (**Fig 8B and 8C**). In contrast, *TLR7* was strongly induced in CD11b^+^ DCs after WNV infection, and on a per cell basis was strongly expressed in all the myeloid subsets except CD11b^-^ DCs.

We conclude that splenic F4/80^hi^ MΦs are especially well programmed to rapidly respond to cytosolic RNA sensing through *Mavs*, whereas MOs, for example, may utilize more signaling through TLRs. Collectively our data suggest that depleting splenic MΦs leads not only to a deficiency in complement components but also to a lesser extent in the RIG-I/MDA5/MAVS pathway, whose components are expressed to a relative lower levels in the other splenic myeloid cell subsets. This could contribute to the susceptibility of splenic MΦ-deficient mice to WNV infection. Furthermore, the major RNA sensor pathways apparently are differentially programmed in splenic myeloid populations. Some of the genes in these pathways change after WNV infection, e.g. *Ddx68* and *Ifih1*, whereas others change very little or only in particular myeloid populations, e.g. *Mavs*, *TLR3*, *TLR7*, and *Myd88*.

## Discussion

Here we have shown that splenic MΦs are required for successful resistance to WNV infection and that splenic MΦs most likely contribute to resistance by sensing WNV RNA and restricting viral spread. Previous studies have shown that MΦs contribute to resistance to WNV infection, but just how MΦs affect WNV infection and whether MΦs in the spleen are essential for protective immunity have been less clear. Systemic depletion of all MΦs and monocytes with CLL increases susceptibility to WNV and viral loads after infection [17]. Local depletion of subcapsular sinus (SCS) MΦs in LNs prior to footpad inoculation of WNV also increased susceptibility to WNV and serum and brain virus levels but did not affect B cell responses significantly [18]. Furthermore, depletion of LN MΦs led to increased and more rapid dissemination of WNV single-cycle viral particles while having no effect on CD8 T cell responses [19]. This suggests that, as is the case for vesicular stomatitis virus [37], SCS MΦs play a major role in preventing the dissemination of WNV. Thus far several studies have shown that local depletion of LN MΦs does not affect antibody responses to viral infections [30,38]. Similarly, our results demonstrate that selective depletion of splenic MΦs does not significantly affect either humoral or T cell immunity to WNV after infection.

Whether or not depletion of splenic MΦs would affect humoral immunity to WNV was unclear since MΦ subsets are functionally distinct, and some MΦ subsets do play key roles in B cell responses [39]. For instance, some splenic MΦs, most likely MZ MΦs, are essential for T cell-dependent Ab responses to particulate bacterial Ags [40]. Within the spleen phenotypically distinct MΦs have been identified in the red pulp, outer MZ and white pulp [39,41]. Furthermore, known receptors for flaviviruses such as MDL-1 and mannose receptors and CLEC5A are differentially expressed on splenic MΦs [42–44]. Splenic MΦs can replicate flaviviruses after taking up the virus directly or with the help of Ab or complement [45,46]. Nevertheless, global depletion of splenic MΦs did not affect adaptive immune responses to WNV. These findings are similar to Seiler et al. [47], who found that while splenic MΦs are essential for preventing spread of lymphocyte choriomeningitis virus, they are not required for generation of CTL responses.

The selective depletion of splenic MΦs, while leaving LN MΦs intact, led mice to become susceptible to WNV and succumb within 12 days of infection. This course was slower than that seen in mice with profound innate immune defects such as IFNAR^-/-^, MAVS^-/-^ or RIG-I^-/-^MDA5^-/-^ mice that die in <8 days [11,48,49]. Rather the rate of death was similar to that seen in mice deficient in complement components or adaptive immune cells such as B cells or CD8 T cells [31,36,50,51]. The humoral immune response in splenic MΦ-depleted mice was robust and was generated early during WNV infection, and sera from WNV-infected splenic MΦ-depleted mice was capable of protecting otherwise susceptible B cell-deficient mice (**Fig 3**). Yet the high level of virus specific Ab was unable to control virus replication occurring in the spleen or prevent spread to the CNS. As the infection progressed, the level of WNV specific IgG and nAb became more similar to that of controls, even though WNV levels were increased in the spleen and brain. Similarly, WNV-specific CD4 and CD8 T cell responses in the splenic MΦ-depleted mice were robust (**Fig 4**), and the brains of WNV-infected splenic MΦ-deficient mice had significant numbers of immune cells (**Fig 5**). Apparently, the adaptive immune responses, while functional in the splenic MΦ-deficient mice, simply could not keep up with the accelerated infection.

The fact that WNV was significantly elevated in both the sera and spleen of splenic MΦ-depleted mice as early as 2 days p.i. suggests that splenic MΦs play a key role early on in preventing WNV spread to other cells in the spleen and elsewhere. Indeed, in the absence of splenic MΦs, after WNV infection very high levels of WNV were detected particularly in splenic CD11c^+^CD11b^-^ DCs and MOs and in MΦs that had repopulated the spleen after MΦ depletion (**Fig 6**) and was detected even in Nphs. In infected control mice little or no WNV was detectable in splenic MOs or Nphs. Apparently, splenic MΦs normally prevent the spread of WNV to MOs and Nphs that otherwise can be infected. The increased numbers of infected MOs in splenic MΦs-depleted mice is noteworthy as they may efficiently deliver WNV to the brain as well as potentially contribute to the accelerated WNV encephalitis in these mice [52,53]. Also, WNV-infected Nphs may contribute to pathogenesis by transporting virus to the CNS [54, 55].

It has been unclear which splenic cells after WNV infection are most responsible for efficiently producing WNV and furthering viral spread [56]. Our data demonstrate that after WNV infection of WT mice, splenic CD11b^-^ DCs express significantly more WNV than splenic MΦs, monocytes, CD11b^+^ DCs or Nphs (**Fig 6A**). One reason this DC subset may produce more WNV is that they express relatively low levels of the RIG-I and MDA5 sensors and thus may be relatively ineffective at activating innate immune protection against WNV. Previous studies have revealed that CD11b^-^CD8α^+^ DCs play a key role in the priming of CD8^+^ cytotoxic T cells to viruses [57]. Furthermore, BATF3^-/-^ mice that do not develop resident CD11b^-^CD8α^+^ DCs or migratory CD11b^-^CD103^+^ DCs have defective virus-specific T cell responses after WNV infection or influenza infections [58–60]. Thus, WNV may target a DC subset that is essential for its control.

The presence of resident MΦs may facilitate the ability of splenic MOs and Nphs to control virus replication. Viral replication in MO populations has been shown to change depending on which host cells first replicate the virus or on viral strains [61, 62]. Ultimately the ability of MOs to control viral replication is dictated by their ability to mount an effective Type I IFN response vs. lower induction of pro-inflammatory cytokines. It is possible that the absence of splenic MΦs skews this balance toward increased viral replication in MOs populations.

Previous studies have implicated innate immune genes in the control of WNV tissue tropism [63]. To determine how splenic MΦs might reduce the spread of WNV, we compared the expression of a set of innate immune-associated genes in purified splenic MΦs, CD11b^-^ DCs, CD11b^+^ DC and MOs in both naïve mice and in PBSL-control mice 4 days after WNV infection. *C1q* genes were selectively expressed in splenic MΦs and *C4b* was dramatically upregulated after WNV infections (**Fig 7, Tables B and C in S1 File**). Both C4^-/-^ and C1q^-/-^ mice succumb to WNV infection [36]; our results suggest that splenic MΦs may be a key source of C4 and C1q that protects against lethal infection. The receptors for complement expressed on MΦs are known to normally facilitate uptake and replication of WNV [45]. Also noteworthy is our finding that Caspase 12 expression both before and after WNV infection was restricted to splenic MΦs and not detected in other splenic myeloid subsets. Caspase 12-deficient mice are highly susceptible to WNV infection, and like splenic MΦ-deficient mice die about 10–12 days after infection [64]. Caspase 12 may function to promote anti-WNV responses by promoting TRIM24 ubiquitination of RIG-I that is essential for RIG-I to mediate type I interferon responses. A deficiency in Caspase 12 and combined with lower levels of splenic RIG-1, thus, may contribute to the susceptibility of splenic MΦ-deficient mice to WNV.

Also, most of *IL18* expression in uninfected mice was restricted to splenic MΦs while after infection splenic MOs expressed high levels of the *IL18* gene. IL18 plays a key non-redundant role the generation of virus-specific NK cells [65], and NK cells may contribute to resistance to WNV [66]. Finally, other immunoregulatory cytokines like IL-10 and IL-33 that play important roles in the control of viral infections are highly expressed by MΦs but not by other myeloid subsets, particularly after WNV infection. In the early phase of antiviral innate immunity IL-10 counterbalances pro-inflammatory signals and protects from tissue damage [67]. IL-33 is essential to attenuate viral-encephalitis by downregulating iNOS expression in the CNS [68]. The absence of these protective anti-inflammatory signals in splenic MΦ-deficient mice may contribute to the increased WNV encephalitis in these mice. While additional studies are required to define further what factors produced by splenic MΦs are essential for anti-flaviviral immunity, our studies underscore how important this myeloid subset is for resistance to WNV.

## Supporting information

S1 File **Fig A. Gating strategies for quantifying splenic and dLN cell subsets.** Splenic and dLN cell suspensions were analyzed by flow cytometry. Debris and doublets were excluded based on scatter profile. Cells were stained for CD11b expression and other markers. **(a)** Spleen cells were identified as follows: CD11b^hi^ F480^hi^ MΦs; F480^-^CD11c^hi^MHCclassII^hi^ DCs further subdivided into CD11b^-^ DCs and CD11b^+^ DCs; NK1.1^+^CD11b^+^ NK cells further subdivided into CD3^-^ NK cells and CD3^+^ NKT cells; CD11b+CD19/CD3^-^ cells subdivided based on side scatter (SSC) and relative Ly6C and Ly6G expression into SSC^lo^Ly6C^lo^ MOs, SSC^lo^Ly6C^hi^ MOs, SSC^hi^Ly6C^+^Ly6G^+^ eosinophils and SSC^hi^Ly6C^+^Ly6G^++^ Nphs; CD11b^-^CD3/CD19^++^ cells further subdivided into MHC class II^++^ B cells and MHC class II^-^ T cells; **(b)** CD19/CD3^++^ cells were excluded and remaining cells analyzed for NK1.1^++^FSC^lo^ NK cells; CD11b^hi^Ly6G^hi^ Nphs; after exclusion of NK cells and Nphs, cells were subdivided based on CD11b, CD11c, F4/80 and Ly6C into DC, MΦ and MO subsets; quantification of NK, NKT, T and B cells in spleens **(c)** and dLNs **(d)** from naïve (black), PBSL-treated (grey) and CLL-treated mice 3 (white) days post-treatment. Statistics: Tukey’s multiple comparison test; * p<0.05, **** p<0.001 **Fig B. Spleen cell population numbers post-WNV infection.** Mice were treated with CLL (open bar) or PBSL (black bar), 3 days prior to s.c. viral (WNV, 1000 PFU) inoculation (footpad), spleens were harvested at day 8 post-WNV. Splenocytes from naïve mice served as a negative control (grey bars). The frequency of myeloid and lymphocyte populations in the spleen were determined by flow cytometry and applied to total splenocytes counts to determine cell numbers for each population. The results shown are the combined result of five experiments. Statistics shown are for Two-tailed Student's t test, * p < 0.05, ** p <0.01, *** p<0.001. **Table A. List of primers for the immune-associated genes tested in the microfluidic qPCR Array Table B. Relative expression of immune-associated genes in splenic myeloid subsets isolated from naïve mice Table C. Relative expression of immune associated genes in splenic myeloid subsets isolated 4 days post-WNV infection**.
(DOCX)
