# Supplementary material for: Splenic macrophages are required for protective innate immunity against West Nile virus
Source: PLoS One. 2018 Feb 6;13(2):e0191690. doi: 10.1371/journal.pone.0191690 (PMC5800658; doi:10.1371/journal.pone.0191690)
Supplement: S1 File — Fig A. Gating strategies for quantifying splenic and dLN cell subsets. Splenic and dLN cell suspensions were analyzed by flow cytometry. Debris and doublets were excluded based on scatter profile. Cells were stained for CD11b expression and other markers. (a) Spleen cells were identified as follows: CD11bhi F480hi MΦs; F480-CD11chiMHCclassIIhi DCs further subdivided into CD11b- DCs and CD11b+ DCs; NK1.1+CD11b+ NK cells further subdivided into CD3- NK cells and CD3+ NKT cells; CD11b+CD19/CD3- cells subdivided based on side scatter (SSC) and relative Ly6C and Ly6G expression into SSCloLy6Clo MOs, SSCloLy6Chi MOs, SSChiLy6C+Ly6G+ eosinophils and SSChiLy6C+Ly6G++ Nphs; CD11b-CD3/CD19++ cells further subdivided into MHC class II++ B cells and MHC class II- T cells; (b) CD19/CD3++ cells were excluded and remaining cells analyzed for NK1.1++FSClo NK cells; CD11bhiLy6Ghi Nphs; after exclusion of NK cells and Nphs, cells were subdivided based on CD11b, CD11c, F4/80 and Ly6C into DC, MΦ and MO subsets; quantification of NK, NKT, T and B cells in spleens (c) and dLNs (d) from naïve (black), PBSL-treated (grey) and CLL-treated mice 3 (white) days post-treatment. Statistics: Tukey’s multiple comparison test; * p<0.05, **** p<0.001 Fig B. Spleen cell population numbers post-WNV infection. Mice were treated with CLL (open bar) or PBSL (black bar), 3 days prior to s.c. viral (WNV, 1000 PFU) inoculation (footpad), spleens were harvested at day 8 post-WNV. Splenocytes from naïve mice served as a negative control (grey bars). The frequency of myeloid and lymphocyte populations in the spleen were determined by flow cytometry and applied to total splenocytes counts to determine cell numbers for each population. The results shown are the combined result of five experiments. Statistics shown are for Two-tailed Student's t test, * p < 0.05, ** p <0.01, *** p<0.001. Table A. List of primers for the immune-associated genes tested in the microfluidic qPCR Array Table B. Relative expression of [file pone.0191690.s001.docx]

**Supporting information Bryan et al. PLOS One**

**
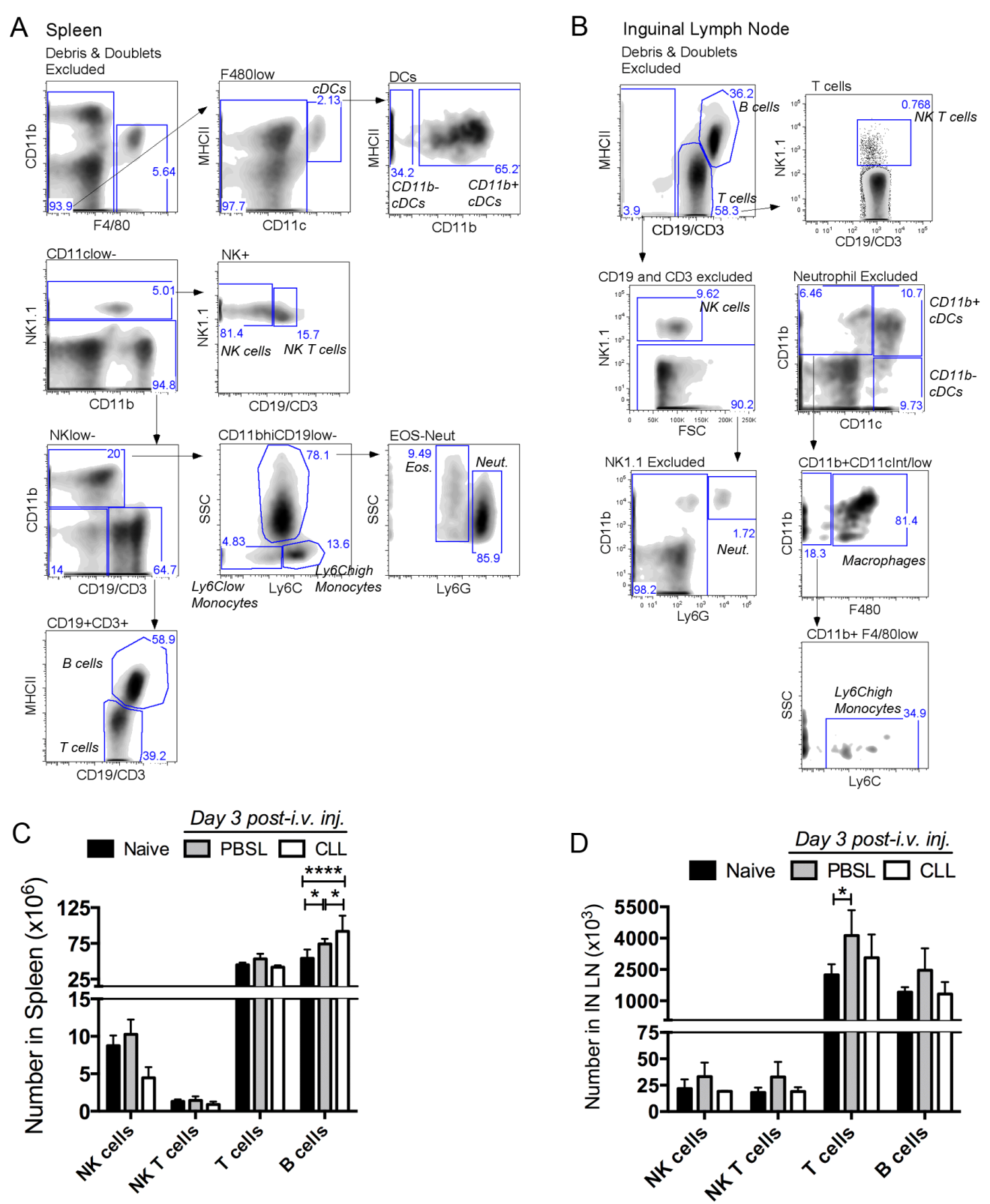
S1 Fig A**

**S1 Fig A. Gating strategies for quantifying splenic and dLN cell subsets.** Splenic and dLN cell suspensions were analyzed by flow cytometry. Debris and doublets were excluded based on scatter profile. Cells were stained for CD11b expression and other markers. **(a)** Spleen cells were identified as follows: CD11b^hi^ F480^hi^  MΦs; F480^-^CD11c^hi^MHCclassII^hi^ DCs further subdivided into CD11b^-^ DCs and CD11b^+^ DCs; NK1.1^+^CD11b^+^ NK cells further subdivided into CD3^-^ NK cells and CD3^+^ NKT cells; CD11b+CD19/CD3^-^ cells subdivided based on side scatter (SSC) and relative Ly6C and Ly6G expression into SSC^lo^Ly6C^lo^ MOs, SSC^lo^Ly6C^hi^ MOs, SSC^hi^Ly6C^+^Ly6G^+^ eosinophils and SSC^hi^Ly6C^+^Ly6G^++^ Nphs; CD11b^-^CD3/CD19^++^ cells further subdivided into MHC class II^++^ B cells and MHC class II^-^ T cells; **(b)** CD19/CD3^++^ cells were excluded and remaining cells analyzed for NK1.1^++^FSC^lo^ NK cells; CD11b^hi^Ly6G^hi^ Nphs; after exclusion of NK cells and Nphs, cells were subdivided based on CD11b, CD11c, F4/80 and Ly6C into DC, MΦ and MO subsets; quantification of NK, NKT, T and B cells in spleens **(c)** and dLNs **(d)** from naïve (black), PBSL-treated (grey) and CLL-treated mice 3 (white) days post-treatment. Statistics: Tukey’s multiple comparison test; * p<0.05, **** p<0.001

**S2 Fig. B**


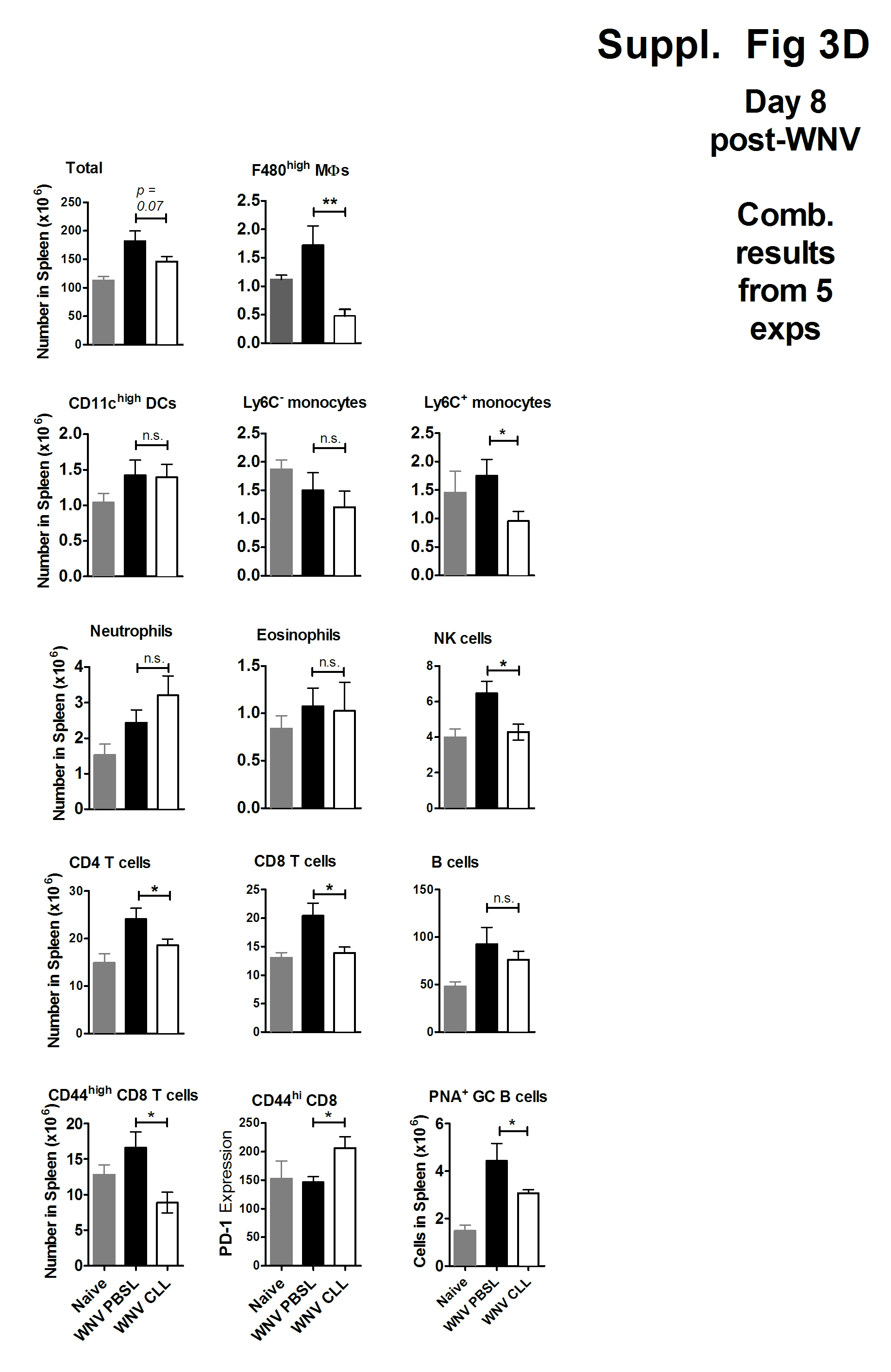


**S1 Fig B. Spleen cell population numbers post-WNV infection.** Mice were treated with CLL (open bar) or PBSL (black bar), 3 days prior to s.c. viral (WNV, 1000 PFU) inoculation (footpad), spleens were harvested at day 8 post-WNV. Splenocytes from naïve mice served as a negative control (grey bars). The frequency of myeloid and lymphocyte populations in the spleen were determined by flow cytometry and applied to total splenocytes counts to determine cell numbers for each population. The results shown are the combined result of five experiments. Statistics shown are for Two-tailed Student's t test, * p < 0.05, ** p <0.01, *** p<0.001.

**S1 Table A. List of primers for the immune-associated genes tested in the microfluidic qPCR Array**

| **Function** | **Gene** | **Primer/probe set from ThermoFisher** |
| --- | --- | --- |
|  |  | **TaqMan® Array Micro Fluidic Cards^a^** |
| **Mph specific (heme induced)** | **Spic** | **Spic-Mm00488428_m1** |
| **Mph specific (MR)** | **Mrc1** | **Mrc1-Mm00485148_m1** |
| **Mph-specific (F480)** | **Emr1** | **Emr1-Mm00802529_m1** |
| **Mph-specific (Heme Scvgr)** | **Cd163** | **Cd163-Mm00474091_m1** |
| **C-type lectin Receptor** | **Clec4n** | **Clec4n-Mm00490931_m1** |
| **Apoptotic cell clearance (phagocytosis)** | **Mertk** | **Mertk-Mm00434920_m1** |
| **Apoptotic cell clearance** | **Grk6** | **Grk6-Mm00442425_m1** |
| **Complement** | **C1qa** | **C1qa-Mm00432142_m1** |
| **Complement** | **C1qb** | **C1qb-Mm01179619_m1** |
| **Complement** | **C1qc** | **C1qc-Mm00776126_m1** |
| **Complement** | **Cfb** | **Cfb-Mm00433909_m1** |
| **Complement** | **C4b** | **C4b-Mm00437893_g1** |
| **Chemokine** | **Cxcl10** | **Cxcl10-Mm00445235_m1** |
| **Chemokine** | **Ccl5** | **Ccl5-Mm01302427_m1** |
| **Cytokine (regulatory)** | **Il10** | **Il10-Mm00439614_m1** |
| **Cytokine Inflammatory** | **Il33** | **Il33-Mm00505403_m1** |
| **Cytokine (regulatory)** | **Tgfb2** | **Tgfb2-Mm00436955_m1** |
| **Receptor (Cytokine)** | **Tgfbr2** | **Tgfbr2-Mm00436977_m1** |
| **Cytokine Inflammatory** | **Il6** | **Il6-Mm00446190_m1** |
| **Cytokine Inflammatory** | **Il23a** | **Il23a-Mm01160011_g1** |
| **Cytokine Inflammatory** | **Tnf** | **Tnf-Mm00443260_g1** |
| **ROS/NOS** | **Nos2** | **Nos2-Mm00440502_m1** |
| **cytokine / inflammasome response** | **Il18** | **Il18-Mm00434225_m1** |
| **cytokine / inflammasome response** | **Il1b** | **Il1b-Mm00434228_m1** |
| **Inflammasome** | **Nlrp3** | **Nlrp3-Mm00840904_m1** |
| **Inflammasome** | **Aim2** | **Aim2-Mm01295719_m1** |
| **Inflammasome** | **Casp1** | **Casp1-Mm00438023_m1** |
| **Inflammasome** | **Nod1** | **Nod1-Mm00805062_m1** |
| **Inflammasome** | **Casp12** | **Casp12-Mm00438038_m1** |
| **Death pathway** | **Casp3** | **Casp3-Mm01195085_m1** |
| **Immune complex uptake FcR ITAM** | **Fcgr1** | **Fcgr1-Mm00438874_m1** |
| **Immune complex uptake FcR ITIM** | **Fcgr2b** | **Fcgr2b-Mm00438875_m1** |
| **Immune complex uptake FcR ITAM** | **Fcgr3** | **Fcgr3-Mm00438882_m1** |
| **Immune complex uptake FcR ITAM** | **Fcgr4** | **Fcgr4-Mm00519988_m1** |
| **TLR (RNA)** | **Tlr3** | **Tlr3-Mm01207404_m1** |
| **TLR (RNA)** | **Tlr7** | **Tlr7-Mm00446590_m1** |
| **TLR (RNA)** | **Tlr8** | **Tlr8-Mm04209873_m1** |
| **TLR** | **Tlr9** | **Tlr9-Mm00446193_m1** |
| **TLR signaling** | **Myd88** | **Myd88-Mm00440338_m1** |
| **Viral sensing (RIGI)** | **Ddx58** | **Ddx58-Mm00554529_m1** |
| **Viral sensing (MDA5)** | **Ifih1** | **Ifih1-Mm00459183_m1** |
| **Viral sensing** | **Mavs** | **Mavs-Mm00523170_m1** |
| **Viral sensing (PKR)** | **Eif2ak2** | **Eif2ak2-Mm01235643_m1** |
| **interferon receptor** | **Ifngr1** | **Ifngr1-Mm00599890_m1** |
| **interferon receptor** | **Ifnar1** | **Ifnar1-Mm00439544_m1** |
| **interferon receptor** | **Ifnar2** | **Ifnar2-Mm00494916_m1** |
| **Interferon response** | **Irf5** | **Irf5-Mm00496477_m1** |
| **Interferon response** | **Irf7** | **Irf7-Mm00516793_g1** |
| **Interferon response** | **Mx1** | **Mx1-Mm00487796_m1** |
| **Interferon response (STING)** | **Tmem173** | **Tmem173-Mm01158117_m1** |
| **Interferon response (susceptibility gene)** | **Oas1b** | **Oas1b-Mm00449297_m1** |
| **T cell activation** | **Cd86** | **Cd86-Mm00444543_m1** |
| **T cell regulation(PDL-1)** | **Cd274** | **Cd274-Mm00452054_m1** |
| **C-type lectin Receptor** | **Clec2d** | **Clec2d-Mm00474134_m1** |
| **C-type lectin Receptor** | **Clec9a** | **Clec9a-Mm00554956_m1** |
| **Interferon response** | **Irf8** | **Irf8-Mm00492567_m1** |
| **Interferon response** | **Irf9** | **Irf9-Mm00492679_m1** |
| **Signaling** | **Stat1** | **Stat1-Mm00439531_m1** |
| **Signaling** | **Stat2** | **Stat2-Mm00490880_m1** |
| Cell signaling, death/survival | Sphk1 | Sphk1-Mm00448841_g1 |
| Complement | Cfd | Cfd-Mm01143935_g1 |
| Complement | C1q(a-c) | C1q(a-c) |
| Cytokine (regulatory) | Tgfb1 | Tgfb1-Mm01178820_m1 |
| Cytokine (regulatory) | Tgfb3 | Tgfb3-Mm00436960_m1 |
| cytokine / interferon response | Ifng | Ifng-Mm01168134_m1 |
| cytokine / interferon response | Ifnb1 | Ifnb1-Mm00439552_s1 |
| Cytokine Inflammatory | Tnfaip3 | Tnfaip3-Mm00437121_m1 |
| Cytokine Inflammatory | Il12b | Il12b-Mm00434174_m1 |
| Cytokine Inflammatory | Il27 | Il27-Mm00461162_m1 |
| Cytokine Inflammatory | Il12a | Il12a-Mm00434165_m1 |
| DC regulation (thrombospondin) | Thbs1 | Thbs1-Mm00449032_g1 |
| Housekeeping gene | Hprt | Hprt-Mm01545399_m1 |
| Housekeeping gene | 18S | 18S-Hs99999901_s1 |
| IFN-independent WNV induced gene | Ppp1r15a | Ppp1r15a-Mm00435119_m1 |
| Inflammasome | Nod2 | Nod2-Mm00467543_m1 |
| Inflammasome | Pycard | Pycard-Mm00445747_g1 |
| Interferon response | Irf1 | Irf1-Mm01288580_m1 |
| Interferon response | Irf3 | Irf3-Mm00516784_m1 |
| Interferon response (TLR3 & MAVS) | Traf3 | Traf3-Mm00495752_m1 |
| MAVS independent response to ER stress | Ddit3 | Ddit3-Mm01135937_g1 |
| Metabolis, decrease with infection? | Pparg | Pparg-Mm01184322_m1 |
| Receptor (BAFFR) | Tnfrsf13c | Tnfrsf13c-Mm00840578_g1 |
| Receptor (Inhibitory) | Sirpa | Sirpa-Mm00455928_m1 |
| Signaling | Stat3 | Stat3-Mm01219775_m1 |
| Signaling (Inhibits JAK-->Stat1) | Socs1 | Socs1-Mm00782550_s1 |
| T cell activation | H2-Ab1 | H2-Ab1-Mm00439216_m1 |
| T cell activation | Cd80 | Cd80-Mm00711660_m1 |
| T cell activation | Cd40 | Cd40-Mm00441891_m1 |
| T cell regulation | Ido2 | Ido2-Mm00524206_m1 |
| T cell regulation | Cd200 | Cd200-Mm00487740_m1 |
| T cell stimulation (OX40) | Tnfrsf4 | Tnfrsf4-Mm00442037_g1 |
| TLR | Tlr4 | Tlr4-Mm00445273_m1 |
| TLR | Tlr2 | Tlr2-Mm00442346_m1 |
| TLR | Tlr5 | Tlr5-Mm00546288_s1 |
| TLR | Tlr13 | Tlr13-Mm01233819_m1 |
| TLR signaling | Tirap | Tirap-Mm00446502_m1 |
| TLR signaling | Ticam2 | Ticam2-Mm01260003_m1 |

^a^ Bold highlights indicate genes shown in Figure 8 and S2 Table and S3 Table.

**S1 Table B. Relative expression of immune-associated genes in splenic myeloid subsets isolated from naïve mice**

|  |  |  | **% total expression^b^ in:** | | | |
| --- | --- | --- | --- | --- | --- | --- |
| **Gene** | **Function** | **Naïve - total expression^a^** | **MΦs** | **CD11b- DCs** | **CD11b+ DCs** | **MOs** |
| **Spic** | **Macrophage** | 89843 | **98.9** | 0.4 | 0.5 | 0.1 |
| **Mrc1** | **Associated** | 142375 | **97.2** | 0.2 | 0.3 | 2.4 |
| **Emr1 (F4/80)** |  | 52462 | **86.9** | 0.4 | 2.5 | 10.2 |
| **CD163** |  | 83634 | **99.5** | 0.1 | 0.3 | 0.0 |
| **Clec4n (Dectin2)** |  | 45986 | **93.8** | 0.4 | 0.8 | 5.0 |
| **Mertk** | **Apoptotic** | 96700 | **99.7** | 0.1 | 0.1 | 0.0 |
| **Grk6** | **Clearance** | 47265 | 17.0 | 2.7 | 3.6 | **76.7** |
| **C1qa** | **Complement** | 259217 | **98.2** | 0.6 | 1.0 | 0.3 |
| **C1qb** |  | 417336 | **99.0** | 0.3 | 0.7 | 0.0 |
| **C1qc** |  | 142212 | **98.9** | 0.5 | 0.5 | 0.1 |
| **Cfb** |  | 2849 | 0.0 | 0.4 | 13.8 | **85.7** |
| **C4b** |  | 128 | 0.0 | 11.5 | 35.2 | **53.3** |
| **Cxcl10** | **Chemokine** | 12300 | **55.6** | 21.2 | 3.8 | 19.4 |
| **Ccl5** |  | 23046 | 0.0 | 12.8 | 36.8 | **50.4** |
| **Il10** | **Regulatory** | 251 | **62.7** | 2.5 | 27.0 | 7.9 |
| **Il33** | **Cytokine/Receptor** | 142 | **79.2** | 1.3 | 5.6 | 14.0 |
| **Tgfb2** |  | 75 | **65.3** | 2.4 | 5.8 | 26.6 |
| **Tgfbr2** |  | 17861 | 0.0 | 6.6 | 8.0 | **85.4** |
| **Il6** | **Inflammatory** | 24476 | 0.6 | 13.9 | 0.0 | **85.5** |
| **Il23a** | **Cytokine** | 22049 | 15.8 | 2.2 | 3.4 | **78.5** |
| **TNFa** |  | 4976 | 18.8 | 13.2 | 11.2 | **56.8** |
| **Nos2** |  | 23 | 0.0 | 7.6 | 7.5 | **84.9** |
| **Il18** | **Inflam/ Cytokine** | 20017 | **91.6** | 2.9 | 2.7 | 2.7 |
| **Il1b** | **/Inflammasome** | 309123 | 1.3 | 0.3 | 0.6 | **97.8** |
| **Nlrp3** | **Inflammasome** | 31383 | 9.9 | 1.5 | 3.8 | **84.8** |
| **Aim2** |  | 12577 | 32.4 | 8.9 | 5.7 | **53.0** |
| **Casp1** |  | 16330 | 0.0 | 5.5 | 24.7 | **69.8** |
| **Nod1** |  | 24706 | **53.3** | 2.2 | 8.5 | 36.0 |
| **Casp12** |  | 26758 | **99.9** | 0.02 | 0.05 | 0.1 |
| **Casp3** | **Death pathway** | 12682 | 0.0 | 7.5 | 8.9 | **83.6** |
| **Fcgr1** | **Fcγ Receptors** | 46581 | **84.3** | 0.2 | 0.4 | 15.1 |
| **Fcgr2b** |  | 17496 | 7.2 | 4.3 | 5.8 | **82.6** |
| **Fcgr3** |  | 88033 | 33.1 | 0.2 | 0.4 | **66.3** |
| **Fcgr4** |  | 34100 | 41.1 | 0.5 | 0.6 | **57.8** |
| **Tlr3** | **Viral RNA** | 10184 | 17.2 | **65.5** | 10.9 | 6.5 |
| **Tlr7** | **Sensing** | 18796 | 49.5 | 1.0 | 4.6 | 45.0 |
| **Tlr8** |  | 35888 | **65.2** | 0.1 | 0.4 | 34.3 |
| **Tlr9** |  | 13780 | 38.4 | 12.8 | 16.4 | 32.4 |
| **Myd88** |  | 44804 | 13.1 | 4.6 | 6.4 | **75.8** |
| **Ddx58 (RIGI)** |  | 16563 | 39.0 | 0.7 | 6.3 | **54.0** |
| **Ifih1 (MDA5)** |  | 8177 | **57.2** | 2.3 | 11.2 | 29.3 |
| **Mavs** |  | 11244 | **70.1** | 3.4 | 5.5 | 21.0 |
| **Eif2ak2 (PKR)** |  | 2650 | 0.0 | 14.9 | 11.6 | **73.5** |
| **Ifngr1** | **Type II IFN associated** | 82647 | 41.6 | 13.7 | 12.5 | 32.2 |
| **Ifnar1** | **Type I IFN** | 22482 | 32.0 | 6.1 | 9.6 | **52.3** |
| **Ifnar2** | **associated** | 59322 | **56.5** | 3.5 | 8.1 | 32.0 |
| **Irf5** |  | 44792 | 25.6 | 11.9 | 15.5 | 47.1 |
| **Irf7** |  | 313717 | 4.6 | 0.7 | 0.6 | **94.0** |
| **Mx1** |  | 5216 | 24.3 | 12.9 | 12.1 | **50.7** |
| **Tmem173 (STING)** |  | 4497 | 41.4 | 13.5 | 28.1 | 17.0 |
| **Oas1b** |  | 1164 | 18.1 | 0.4 | 8.4 | **73.1** |
| **Cd86** | **Other Receptors** | 33937 | **83.2** | 8.2 | 4.1 | 4.5 |
| **Cd274** |  | 10803 | 32.1 | 3.5 | 25.3 | 39.1 |
| **Clec2d** |  | 32175 | 38.5 | 7.2 | 13.6 | 40.7 |
| **Clec9a** |  | 11930 | 19.1 | **59.8** | 13.9 | 7.2 |
| **Irf8** | **Interferon** | 54883 | 0.0 | **64.2** | 18.4 | 17.4 |
| **Irf9** | **response /** | 21089 | 31.1 | 7.2 | 10.4 | **51.3** |
| **Stat1** | **Signaling** | 70427 | **57.0** | 3.6 | 4.8 | 34.6 |
| **Stat2** |  | 22014 | **51.9** | 7.7 | 12.9 | 27.5 |

^a^Spleen cells were obtained from PBSL-treated control naïve mice and myeloid cell subsets isolated using flow cytometry (see Materials and Methods). RNA was extracted from each subset, converted to cDNA and analyzed for gene expression by qPCR. Gene expression refers to the 2ˆ(-dCT) value generated using DataAssist3.1 with 18s and HPRT as endogenous controls. Indicated is the total gene expression for all myeloid populations.

^b^ Gene expression per population for the four subsets relative to the total gene expression in myeloid cells. Gene expression per population shown is calculated as gene expression of the given population multiplied by the frequency of the same population in the spleen. Bold highlights indicate genes that are expressed ≥ 50% in one myeloid subset compared to the other subsets.

**S1 Table C. Relative expression of immune associated genes in splenic myeloid subsets isolated 4 days post-WNV infection. Genes significantly increased in total splenic myeloid cells**

|  |  |  |  | **% total expression^b^ in:** | | | |
| --- | --- | --- | --- | --- | --- | --- | --- |
| **Gene** | **Function** | **WNV D4 -total expression^a^** | **Ratio to total uninfected^c^** | **MΦs** | **CD11b- DCs** | **CD11b+ DCs** | **MOs** |
| **Spic** | **Macrophage** | 207806 | 2.3 | **98.2** | 0.4 | 0.7 | 0.7 |
| **Mrc1 (MR)** | **Associated** | 323354 | 2.3 | **99.3** | 0.2 | 0.1 | 0.4 |
| **Emr1 (F4/80)** |  | 103153 | 2.0 | **88.4** | 0.3 | 3.6 | 7.7 |
| **CD163** |  | 74841 | **0.9** | **99.6** | 0.1 | 0.2 | 0.1 |
| **Clec4n (Dectin 2)** |  | 122047 | 2.7 | **97.4** | 0.2 | 0.6 | 1.8 |
| **Mertk** | **Apoptotic** | 448544 | **4.6** | **99.4** | 0.1 | 0.1 | 0.4 |
| **Grk6** | **Clearance** | 108035 | 2.3 | 17.1 | 3.2 | 4.1 | **75.6** |
| **C1qa** | **Complement** | 1112102 | **4.3** | **99.2** | 0.2 | 0.5 | 0.1 |
| **C1qb** |  | 1099129 | 2.6 | **99.2** | 0.2 | 0.5 | 0.2 |
| **C1qc** |  | 330197 | 2.3 | **99.3** | 0.2 | 0.3 | 0.1 |
| **Cfb** |  | 46969 | **16.5** | 46.5 | 0.4 | 4.2 | 49.0 |
| **C4b** |  | 52328 | **410.1** | **94.3** | 0.1 | 0.1 | 5.5 |
| **Cxcl10** | **Chemokine** | 58233 | **4.7** | 39.4 | 4.2 | 11.3 | 45.2 |
| **Ccl5** |  | 137080 | **5.9** | 2.3 | 7.6 | 37.2 | **52.8** |
| **Il10** | **Regulatory** | 579 | 2.3 | **88.9** | 2.4 | 5.5 | 3.2 |
| **Il33** | **Cytokine/** | 466 | **3.3** | **94.7** | 0.5 | 0.9 | 4.0 |
| **Tgfb2** | **Receptor** | 327 | **4.4** | **91.9** | 1.0 | 1.4 | 5.7 |
| **Tgfbr2** |  | 94445 | **5.3** | **59.7** | 2.3 | 3.6 | 34.4 |
| **Il6** | **Inflammatory** | 6754 | **0.3** | 29.2 | 12.3 | 1.7 | **56.8** |
| **Il23a** | **Cytokine** | 5832 | **0.3** | 33.2 | 6.2 | 29.7 | 30.9 |
| **TNFa** |  | 11785 | 2.4 | 28.5 | 17.1 | 23.3 | 31.2 |
| **Nos2** |  | 2623 | **112.3** | 4.9 | 11.8 | 6.9 | **76.4** |
| **Il18** | **InflamCytokine** | 393901 | 1.3 | 3.2 | 0.4 | 1.1 | **95.3** |
| **Il1b** | **/Inflammasome** | 67610 | **3.4** | **86.1** | 1.4 | 2.4 | 10.1 |
| **Nlrp3** | **Inflammasome** | 44871 | 1.4 | 18.5 | 2.9 | 15.6 | **63.1** |
| **Aim2** |  | 22067 | 1.8 | 29.4 | 16.7 | 22.5 | 31.5 |
| **Casp1** |  | 73227 | **4.5** | 39.1 | 3.8 | 12.0 | 45.0 |
| **Nod1** |  | 81100 | **3.3** | 49.3 | 2.5 | 10.6 | 37.7 |
| **Casp12** |  | 14165 | **0.5** | **97.9** | 0.01 | 0.03 | 2.1 |
| **Casp3** | **Death pathway** | 43658 | **3.4** | 44.7 | 8.0 | 9.2 | 38.1 |
| **Fcgr1** | **Fcγ Receptors** | 321349 | **6.9** | **56.4** | 0.2 | 1.1 | 42.2 |
| **Fcgr2b** |  | 37096 | 2.1 | 12.5 | 4.3 | 6.0 | **77.2** |
| **Fcgr3** |  | 141418 | 1.6 | 42.0 | 0.1 | 0.2 | **57.6** |
| **Fcgr4** |  | 81621 | 2.4 | **72.7** | 0.4 | 1.5 | 25.4 |
| **Tlr3** | **Viral RNA** | 18705 | 1.8 | 36.9 | 53.9 | 7.0 | 2.2 |
| **Tlr7** | **Sensing** | 69014 | **3.7** | **56.2** | 0.8 | 18.6 | 24.3 |
| **Tlr8** |  | 26126 | **0.7** | **76.3** | 0.1 | 0.4 | 23.2 |
| **Tlr9** |  | 66843 | **4.9** | 37.1 | 10.0 | 15.3 | 37.6 |
| **Myd88** |  | 124142 | 2.8 | 16.8 | 4.3 | 7.7 | **71.2** |
| **Ddx58 (RIGI)** |  | 95684 | **5.8** | 33.4 | 1.2 | 10.9 | **54.5** |
| **Ifih1 (MDA5)** |  | 58850 | **7.2** | 39.9 | 3.5 | 16.0 | 40.6 |
| **Mavs** |  | 24850 | 2.2 | **69.0** | 6.3 | 10.2 | 14.5 |
| **Eif2ak2 (PKR)** |  | 30652 | **11.6** | 44.0 | 8.1 | 10.6 | 37.3 |
| **Ifngr1** | **Type II IFN associated** | 148295 | 1.8 | 36.9 | 12.5 | 15.1 | 35.5 |
| **Ifnar1** | **Type I IFN** | 37384 | 1.7 | 32.3 | 9.3 | 16.4 | 42.0 |
| **Ifnar2** | **associated** | 136600 | 2.3 | **55.2** | 5.7 | 11.6 | 27.6 |
| **Irf5** |  | 114869 | 2.6 | 28.7 | 14.7 | 12.0 | 44.6 |
| **Irf7** |  | 1018146 | **3.2** | 45.4 | 3.6 | 6.0 | 44.9 |
| **Mx1** |  | 52185 | **10.0** | 43.0 | 8.9 | 12.9 | 35.2 |
| **Tmem173 (STING)** |  | 15634 | **3.5** | 22.7 | 11.4 | 24.6 | 41.3 |
| **Oas1b** |  | 8037 | **6.9** | 30.8 | 2.6 | 18.7 | 47.9 |
| **Cd86** | **Other** | 70443 | 2.1 | **82.3** | 9.8 | 5.3 | 2.5 |
| **Cd274 (PDL-1)** | **Receptors** | 44184 | **4.1** | 34.5 | 4.3 | 20.6 | 40.6 |
| **Clec2d** |  | 97799 | **3.0** | 45.3 | 5.6 | 9.9 | 39.2 |
| **Clec9a** |  | 37532 | **3.1** | 18.0 | **66.6** | 12.6 | 2.8 |
| **Irf8** | **Interferon** | 184961 | **3.4** | 34.8 | 45.8 | 5.0 | 14.5 |
| **Irf9** | **response /** | 75064 | **3.6** | 21.2 | 11.2 | 15.4 | **52.2** |
| **Stat1** | **Signaling** | 265589 | **3.8** | **50.2** | 6.3 | 9.0 | 34.5 |
| **Stat2** |  | 147025 | **6.7** | 39.1 | 7.3 | 10.5 | 43.1 |

^a^Spleen cells were obtained from PBSL-treated control mice 4 days p.i. WNV infection and myeloid cell subsets isolated using flow cytometry (see Materials and Methods). RNA was extracted from each subset, converted to cDNA and analyzed for gene expression by qPCR. Gene expression refers to the 2ˆ(-dCT) value generated using DataAssist3.1 with 18s and HPRT as endogenous controls. Indicated is the total gene expression for all myeloid populations.

^b^Gene expression per population for the four subsets relative to the total gene expression in myeloid cells. Gene expression per population shown is calculated as gene expression of the given population multiplied by the frequency of the same population in the spleen. Bold highlights indicate genes that are expressed ≥ 50% in one myeloid subset compared to the other subsets.

^c^ Ratio of total gene expression per population in myeloid populations in PBSL-treated mice 4 days p.i. vs. gene expression in naïve mice. Genes that are upregulated ≥ 3-fold are in bold and highlighted in dark grey. Genes that are downregulated are highlighted in light grey.
